# Whole-Genome Resequencing of Worldwide Wild and Domestic Sheep Elucidates Genetic Diversity, Introgression, and Agronomically Important Loci

**DOI:** 10.1093/molbev/msab353

**Published:** 2021-12-10

**Authors:** Feng-Hua Lv, Yin-Hong Cao, Guang-Jian Liu, Ling-Yun Luo, Ran Lu, Ming-Jun Liu, Wen-Rong Li, Ping Zhou, Xin-Hua Wang, Min Shen, Lei Gao, Jing-Quan Yang, Hua Yang, Yong-Lin Yang, Chang-Bin Liu, Peng-Cheng Wan, Yun-Sheng Zhang, Wen-Hui Pi, Yan-Ling Ren, Zhi-Qiang Shen, Feng Wang, Yu-Tao Wang, Jin-Quan Li, Hosein Salehian-Dehkordi, Eer Hehua, Yong-Gang Liu, Jian-Fei Chen, Jian-Kui Wang, Xue-Mei Deng, Ali Esmailizadeh, Mostafa Dehghani-Qanatqestani, Hadi Charati, Maryam Nosrati, Ondřej Štěpánek, Hossam E Rushdi, Ingrid Olsaker, Ino Curik, Neena A Gorkhali, Samuel R Paiva, Alexandre R Caetano, Elena Ciani, Marcel Amills, Christina Weimann, Georg Erhardt, Agraw Amane, Joram M Mwacharo, Jian-Lin Han, Olivier Hanotte, Kathiravan Periasamy, Anna M Johansson, Jón H Hallsson, Juha Kantanen, David W Coltman, Michael W Bruford, Johannes A Lenstra, Meng-Hua Li

**Affiliations:** 1 College of Animal Science and Technology, China Agricultural University, Beijing, China; 2 CAS Key Laboratory of Animal Ecology and Conservation Biology, Institute of Zoology, Chinese Academy of Sciences (CAS), Beijing, China; 3 College of Life Sciences, University of Chinese Academy of Sciences (UCAS), Beijing, China; 4 Novogene Bioinformatics Institute, Beijing, China; 5 Animal Biotechnological Research Center, Xinjiang Academy of Animal Science, Urumqi, China; 6 Institute of Animal Husbandry and Veterinary Medicine, Xinjiang Academy of Agricultural and Reclamation Sciences, Shihezi, China; 7 State Key Laboratory of Sheep Genetic Improvement and Healthy Production, Xinjiang Academy of Agricultural and Reclamation Sciences, Shihezi, China; 8 Shandong Binzhou Academy of Animal Science and Veterinary Medicine, Binzhou, China; 9 Institute of Sheep and Goat Science, Nanjing Agricultural University, Nanjing, China; 10 College of Life and Geographic Sciences, Kashi University, Kashi, China; 11 College of Animal Science, Inner Mongolia Agricultural University, Hohhot, China; 12 Grass-Feeding Livestock Engineering Technology Research Center, Ningxia Academy of Agriculture and Forestry Sciences, Yinchuan, China; 13 College of Animal Science and Technology, Yunnan Agricultural University, Kunming, China; 14 Department of Animal Science, Faculty of Agriculture, Shahid Bahonar University of Kerman, Kerman, Iran; 15 Department of Agriculture, Payame Noor University, Tehran, Iran; 16 Department of Virology, State Veterinary Institute Jihlava, Jihlava, Czech Republic; 17 Department of Animal Production, Faculty of Agriculture, Cairo University, 12613 Giza, Egypt; 18 Department of Preclinical Sciences and Pathology, Faculty of Veterinary Medicine, Norwegian University of Life Sciences, Ås, Norway; 19 Department of Animal Science, Faculty of Agriculture, University of Zagreb, Zagreb, Croatia; 20 Animal Breeding Division, National Animal Science Institute, Nepal Agriculture Research Council (NARC), Kathmandu, Nepal; 21 Embrapa Recursos Genéticos e Biotecnologia, Parque Estação Biológica, PqEB, Brasília, DF, Brazil; 22 Dipartimento di Bioscienze, Biotecnologie e Biofarmaceutica, Università degli Studi di Bari Aldo 24 Moro, Bari, Italy; 23 Department of Animal Genetics, Center for Research in Agricultural Genomics (CRAG), CSIC-IRTA-UAB-UB, Campus de la Universitat Autònoma de Barcelona, Bellaterra, Spain; 24 Department of Animal Sciences, Universitat Autònoma de Barcelona, Bellaterra, Spain; 25 Department of Animal Breeding and Genetics, Justus-Liebig-University Giessen, Giessen, Germany; 26 Department of Microbial, Cellular and Molecular Biology, Addis Ababa University, Addis Ababa, Ethiopia; 27 LiveGene Program, International Livestock Research Institute, Addis Ababa, Ethiopia; 28 Small Ruminant Genomics, International Centre for Agricultural Research in the Dry Areas (ICARDA), Addis Ababa, Ethiopia; 29 CTLGH and SRUC, The Roslin Institute Building, Easter Bush Campus, Edinburgh, Scotland; 30 CAAS-ILRI Joint Laboratory on Livestock and Forage Genetic Resources, Institute of Animal Science, Chinese Academy of Agricultural Sciences (CAAS), Beijing, China; 31 Livestock Genetics Program, International Livestock Research Institute (ILRI), Nairobi, Kenya; 32 School of Life Sciences, University of Nottingham, University Park, Nottingham, United Kingdom; 33 Animal Production and Health Laboratory, Joint FAO/IAEA Division, International Atomic Energy Agency (IAEA), Vienna, Austria; 34 Department of Animal Breeding and Genetics, Faculty of Veterinary Medicine and Animal Science, Swedish University of Agricultural Sciences, Uppsala, Sweden; 35 Faculty of Natural Resources and Environmental Sciences, Agricultural University of Iceland, Borgarnes, Iceland; 36 Production Systems, Natural Resources Institute Finland (Luke), Jokioinen, Finland; 37 Department of Biological Sciences, University of Alberta, Edmonton, Alberta, Canada; 38 School of Biosciences, Cardiff University, Cathays Park, Cardiff, Wales, United Kingdom; 39 Sustainable Places Research Institute, Cardiff University, Wales, United Kingdom; 40 Faculty of Veterinary Medicine, Utrecht University, Utrecht, the Netherlands

**Keywords:** whole-genome sequences, genetic diversity, adaptive introgression, genetic selection, agronomic traits, migration

## Abstract

Domestic sheep and their wild relatives harbor substantial genetic variants that can form the backbone of molecular breeding, but their genome landscapes remain understudied. Here, we present a comprehensive genome resource for wild ovine species, landraces and improved breeds of domestic sheep, comprising high-coverage (∼16.10×) whole genomes of 810 samples from 7 wild species and 158 diverse domestic populations. We detected, in total, ∼121.2 million single nucleotide polymorphisms, ∼61 million of which are novel. Some display significant (*P* < 0.001) differences in frequency between wild and domestic species, or are private to continent-wide or individual sheep populations. Retained or introgressed wild gene variants in domestic populations have contributed to local adaptation, such as the variation in the *HBB* associated with plateau adaptation. We identified novel and previously reported targets of selection on morphological and agronomic traits such as stature, horn, tail configuration, and wool fineness. We explored the genetic basis of wool fineness and unveiled a novel mutation (chr25: T7,068,586C) in the 3′-UTR of *IRF2BP2* as plausible causal variant for fleece fiber diameter. We reconstructed prehistorical migrations from the Near Eastern domestication center to South-and-Southeast Asia and found two main waves of migrations across the Eurasian Steppe and the Iranian Plateau in the Early and Late Bronze Ages. Our findings refine our understanding of genome variation as shaped by continental migrations, introgression, adaptation, and selection of sheep.

## Introduction

Since their domestication, sheep (*Ovis aries*) have been providing meat, wool, skin, milk, and other products to humans. They are essential for welfare of hundreds of millions of people living in rural communities of some Asian, African, and South American countries (Nkedianye et al. 2019). Since their domestication from Asiatic mouflon ∼10,000 years ago in the Near East (Zeder 2008), sheep expanded to other parts of Asia (Lv et al. 2015; Zhao et al. 2017) and to Europe (Ryder 1984; Tapio et al. 2006; Ciani et al. 2020), Africa (Bradford and Fitzhugh 1984; Muigai and Hanotte 2013), and the New World (Crispim et al. 2012; Campos et al. 2020; Revelo et al. 2020; Thorne et al. 2021). An early and major event was the post-domestication transition of hair sheep to wool sheep (Chessa et al. 2009). In addition, introgression from wild relatives into domestic populations has occurred (Barbato et al. 2017; Hu et al. 2019; Ciani et al. 2020; Cao et al. 2021). Thus, sheep have acquired a worldwide distribution through adaptation to a diverse range of environments and genetic improvement under different production systems, developing into plenty of unique breeds (Lv et al. 2015; Cao et al. 2021). Hence, the extensive variations in landraces and improved breeds underpin the genomic variations, adaptive characteristics, and important agronomic traits of domestic sheep (Yang et al. 2016; Li et al. 2020).

**Fig. 1.**
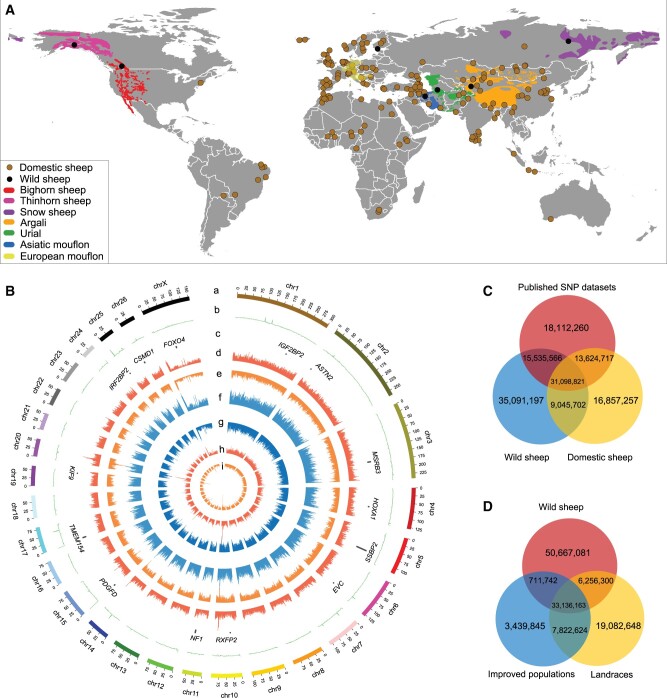
Geographic distributions and genetic variants of wild and domestic sheep. (*A*) Sampling locations of 158 domestic sheep populations, and geographic distribution ranges and sampling locations of the seven wild sheep species. The brown and black dots indicate the sampling locations of domestic and wild sheep. The geographic distribution range of each wild sheep species is shown on the map with different colors as follows: *Ovis musimon* (yellow), *O. orientalis* (blue), *O. vignei* (green), *O. ammon* (orange), *O. nivicola* (purple), *O. dalli* (pink), and *O canadensis* (red). All European mouflon populations in wild parks of Europe have been imported recently from Coasica and Sardinia. (*B*) Diagram of SNPs found by resequencing of 734 individuals. Circles represent from outermost to innermost, 27 chromosomes (chr. 1–26, X) denoted by different colors (a), average sequence depth for 810 individuals (b), candidate genes selection based on the top 1% values of global *F*_ST_ (c), SNP abundance bars in improved populations (d) and landraces (e), nucleotide diversity (*π*) abundance bars in improved populations (f) and landraces (g), and INDEL abundance bars in improved populations (h) and landraces (i). (*C*) Venn diagrams for novel variants detected in wild and domestic sheep. (*D*) Venn diagrams for variants shared among wild sheep species and improved populations and landraces of domestic sheep.

In this study, we sequenced genomes with an average depth of ∼16.7× for one Asiatic mouflon and a diverse set of 165 samples from 82 domestic breeds. These include African, South Asian, Southeast Asian, Central Asian, East Asian, South American, European, and the Middle Eastern breeds which were underrepresented in early work (Alberto et al. 2018; Naval-Sanchez et al. 2018; Deng et al. 2020; Li et al. 2020), but are essential to characterize the genetic variation and the introgression from wild into domestic sheep, and to explore for the first time the history of South-and-Southeast Asian sheep. By combining these novel genomic resources with previously published whole-genome sequences (WGS; >10×), we achieved a total of 810 samples from 158 domestic breeds and seven wild ovine species (fig. 1*A*, supplementary table S1, Supplementary Material online). Using this comprehensive data set, we explored patterns of genome-wide sequence variation among species/breeds, characterized regional and population-specific variants, inferred demographic expansions, discovered retained or introgressed variants from wild sheep, and identified genes and variants associated with agronomically important traits in domestic sheep.

## Results

### Whole-Genome Sequencing and Variation

Individual genomes of one Asiatic mouflon and 165 samples from 82 domestic sheep breeds across the world were sequenced to an average depth of 16.7× and average genome coverage of 96.40% to the *Oar_rambouillet_v1.0* reference genome. These data were combined with 644 available genomes (fig. 1*A* and supplementary table S1, Supplementary Material online). Of the 82 domestic breeds, whole genomes of 24 breeds from regions such as Africa (e.g., Menz), Central-and-East Asia (e.g., Minxian Black Fur, Gala, Azerbaijian Mountain Merino, Kazakh Arkhar-Merino, and Kazakh Edilbai), South-and-Southeast Asia (e.g., Indonesia Thin-tailed, Kajli, Vembur, Mecheri, Madras Red, Pattanam, Kilakarsal, and Baruwal), Europe (e.g., Russian Edilbai, Lleyn, Hebridean, Ryelands, Swedish Finewool, Icelandic Leader, Icelandic, and Oxford Down), and America (e.g., Morada Nova) were sequenced for the first time and extended our coverage of the sheep genetic resources (supplementary table S1, Supplementary Material online).

In total, we analyzed ∼31.5 Tb raw data from 810 samples, comprising ∼41 billion 100-bp and 150-bp paired-end reads with average genome depth of 16.10× per individual (or ∼39.8 Gb raw data per sample). The reads were mapped, resulting in varied alignment rate, genome coverage and sequence depth among individuals, and across the whole genome (fig. 1*B* and supplementary table S1, Supplementary Material online). After filtering, we identified ∼137.7 million genome-wide genetic variants, including ∼121.2 million single nucleotide polymorphisms (SNPs) (∼12.1 million/individual), ∼16.4 million small insertions and deletions (INDELs, <50 bp, ∼15.5 million/individual), and 74,173 structural variants (SVs, 51–886,322 bp, 9,190/individual; 58,256 deletions, 10,890 translocations, 3,501 duplications, and 1,524 inversions) across all the samples (fig. 1*B* and table 1). The number of SNP variants per individual showed significant (Mann–Whitney, *P* < 0.001) difference between species (supplementary table S2, Supplementary Material online). Of the set of ∼121.2 million high-quality SNPs, 77.9% had a minor allele frequency (MAF) < 5% (supplementary fig. S1*A*, Supplementary Material online). The number of SNP identified varies from 9.82 to 12.9 million per domestic individual, ∼9.9 million (European mouflon) to ∼18.46 million (Bighorn) per wild individual (supplementary fig. S1*B*, Supplementary Material online), and 10.4 to 13.0 million per domestic breed, which did not depend on the population sample size (supplementary fig. S1*C*, Supplementary Material online).

**Table 1.** Summary of Whole-Genome Variations Identified in Wild and Domestic Sheep.

Variations		Wild Sheep	Domestic Sheep	Total
SNPs		90,771,286	70,626,497	121,253,260
INDELs		12,304,629	10,694,323	16,409,609
Structural variants	Deletions	42,048	40,885	58,256
	Translocations	4,823	9,315	10,890
	Duplications	1,875	2,742	3,501
	Inversions	1,035	1,064	1,524
	Insertions	0	2	2

We identified ∼61.0 million SNPs (50.2%) that have not been reported previously in the dbSNP (ftp://ftp.ncbi.nih.gov/snp/organisms/archive/sheep_9940/, last accessed July 10, 2021) or the European Variation Archive (EVA, including accessions PRJEB33111, PRJEB23437, PRJEB6025, PRJEB15642, and PRJEB14685) (fig. 1*C*), which could be due to prior underrepresentation of the sheep breeds studied here. We observed ∼4.51 million (∼3.7% of the total SNPs) SNPs shared among all the eight ovine species, ∼5.64 million (European mouflon vs. snow sheep) to 34.6 million (domestic sheep vs. Asiatic mouflon) between pairwise species, ∼50.7 million only in wild species, and ∼40.96 million SNPs shared between landraces and improved breeds of domestic sheep (fig. 1*D*). We identified ∼0.024 million (Brazilian Creole) to ∼1.74 million (local Moroccan sheep) breed-specific SNPs (supplementary fig. S2, Supplementary Material online). The number of SNPs present in each major geographic region, including region-specific SNPs, varies from 27.8 million in the American breeds to ∼49.4 million in the Central-and-East Asian breeds (supplementary fig. S3 and table S3, Supplementary Material online), which could be partially ascribed to the differences in individual WGS variability and number of samples included in the study.

### Variant Accuracy

On average, 9,954,398 of the SNPs (82.05%) identified in domestic sheep and 8,252,754–13,976,424 of the SNPs detected in the wild species (60.26–81.47%) were confirmed in the sheep dbSNP database v.151 (supplementary table S4, Supplementary Material online). The proportions of SNPs confirmed were comparable with those reported in a previous study (Li et al. 2020). In addition, we inspected 133 selected SNPs in candidate functional genes below from 2 to 16 individuals of ten populations obtained by Sanger sequencing approach, giving an overall validation rate of 98.58% (supplementary table S5, Supplementary Material online). Overall, the results provided confidence on the accuracy of variant calling.

### Genome Variant Map

The average SNP densities were 43.67/kb, 33.15/kb, and 19.20/kb in autosomes, X, and Y-chromosomes, respectively, illustrating the notion that the nucleotide diversity was lower on the nonrecombining sex chromosomes (Sayres 2018). From all SNPs, 44.21% were located in or near genes, including upstream (6.71%) and downstream (2.66%) of open reading frame, introns (32.84%) and untranslated regions (UTRs, 0.88%). In total, 1,042,367 SNPs were located in protein-coding exons, causing 492,609 nonsynonymous and 549,128 synonymous mutations, with 45,679 start codon and 8,803 stop codon changes, showing their potentially large impact on function of the relevant genes (supplementary table S6, Supplementary Material online).

From the 26,342 SVs discovered only in domestic sheep, 11,391 (43.24%) were predicted to have functional consequences, and 6,816 (25.88%) are in 3,592 functional genes (e.g., *BMPR1B* and *MYPN*; supplementary table S7, Supplementary Material online). Gene ontology (GO) and Kyoto Encyclopedia of Genes and Genomes (KEGG) annotations of the SVs indicated these genes to be involved in axonogenesis and axon guidance (supplementary fig. S4 and table S8, Supplementary Material online). We found 19,875 SVs present only in the seven wild sheep species. GO and KEGG enrichment analyses for 8,215 genes associated with these unique SVs in wild sheep uncovered their essential roles in MAPK signaling pathway, cell morphogenesis, and GTPase activator activity (supplementary fig. S5 and table S9, Supplementary Material online).

### Genomic Diversity

**Fig. 2.**
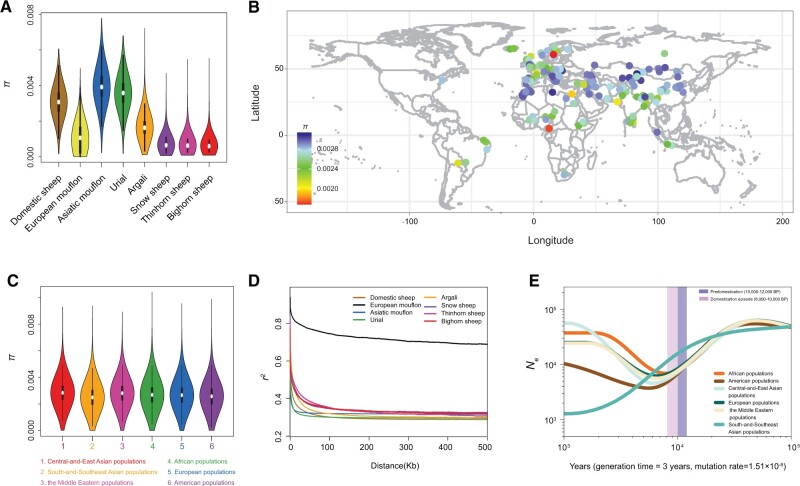
Genetic variability of wild and domestic sheep. (*A*) Average nucleotide diversity (*π*) of eight wild and domestic sheep species. (*B*) Nucleotide diversity (*π*) of 158 domestic populations across the world. (*C*) Average nucleotide diversity (*π*) of six geographic groups of domestic sheep populations. (*D*) Decay of LD for eight wild and domestic sheep species, with one line per species. (*E*) Effective population sizes (*N*_e_) for six domestic sheep populations inferred using SMC++, with one line per population. A generation time of 3 years and mutation rate of 1.51 × 10^−8^ per site per generation were used to convert coalescent scaling to calendar time.

Nucleotide diversities (π) in the eight Ovine species were estimated at individual level after the correction for sample size. Asiatic mouflon and urial have the highest nucleotide diversity, whereas the lowest was observed in bighorn sheep (fig. 2*A*). Asiatic mouflon and urial, which genetically are not completely separated (Demirci et al. 2013; Deng et al. 2020) (see also Results), are potential resources to expand the genetic resources of domestic improvement. The diversity of domestic sheep varies considerably with π values ranging from π = 3.1 × 10^-3^ (Bashibai breed) to 1.7 × 10^-3^ (Svärdsjö breed; fig. 2*B* and supplementary table S10, Supplementary Material online). There are no large differences among breeds from different geographic regions (fig. 2*C*). Breeds with a low diversity show a high coverage by runs of homozygosity (ROH; supplementary fig. S6 and table S10, Supplementary Material online). Sheep from central and northern Europe with a long history of breed formation and selective breeding have a relatively low diversity compared to most other breeds. There are no systematic differences between landraces and productive breeds: several fine-wool breeds and breeds from marginal regions such as the Balkan, the Caucasus, Libya, Kazakhstan, and Tibet and Xinjiang of China have retained high diversity, whereas small population sizes have decreased the diversity in breeds from both categories (fig. 2*B* and supplementary table S10, Supplementary Material online). In general, the values of nucleotide diversity calculated here are in the range of earlier estimates in domestic sheep (π = 1.9–2.5 × 10^-3^, Yang et al. 2016; π = 1.6 × 10^-3^, Naval-Sanchez et al. 2018; π = 2.15–2.68 × 10^-3^, Alberto et al. 2018; π = 2.44–2.84 × 10^-3^, Pan et al. 2018; π = 1.2–42 × 10^-3^, Hu et al. 2019; π = 1.05–1.18 × 10^-3^, Li et al. 2020; π = 3.2 × 10^-3^, Chen et al. 2021) (supplementary table S11, Supplementary Material online). For the same populations such as Merino, Tan, Lop, and Tibetan sheep, most showed similar estimates in this and early studies (supplementary table S12, Supplementary Material online), but the discrepancy in some populations (e.g., Iran sheep) can be due to the difference in sequencing depth, sample size (e.g., the number of animals sequenced) and the sheep reference genomes (*Oar v.4.0* or *Oar_rambouillet_v1.0*) used in the SNP calling, etc.

Linkage disequilibrium (LD) analysis indicated that the physical distance between SNPs measured as half of its maximal value occurred at 40.0 kb (*r*^2^ = 0.35) for domestic sheep and at 29.1–37.7 kb (*r*^2^ = 0.31–0.40) for wild sheep except the inbred European mouflons (fig. 2*D*). For landraces, LD values ranged from 14 to 296 kb (but >1,000 kb for the Cameroon population) and were higher than for improved breeds (14.6–67 kb) (supplementary fig. S7*A* and *B*, Supplementary Material online). The LD decay in domestic sheep was slower than in cattle (∼2–10 kb) (Mei et al. 2018), goat (∼1–2.5 kb) (Zheng et al. 2020), pig (∼ 8–20 kb) (Li et al. 2013), and dog (∼1–3 kb) (Wang et al. 2015).

Global *F*_ST_ value for domestic sheep (*F*_ST_ = 0.082, *P* < 0.001) is lower than those across other domestic species such as goats (*F*_ST_ = 0.112) (Kim et al. 2019) and pigs (*F*_ST_ = 0.115) (Mu**ñ**oz et al. 2019), which may partially reflect a relatively frequent genetic exchange during the development of modern breeds (Kijas et al. 2012). *F*_ST_ value for breeds from the same region ranges from 0.050 (the Middle East) to 0.193 (America; supplementary fig. S8, Supplementary Material online).

### Effective Population Size

Pairwise sequentially Markovian coalescent (PSMC) analysis revealed that all wild and domestic sheep species experienced a dramatic contraction in *N*_e_ ∼100–400 thousand years ago (ka). Ancestors of domestic sheep (i.e., Asiatic mouflon) underwent a second decline in *N*_e_ during ∼10,000–30,000 years BP, coinciding with the glacial periods (supplementary fig. S9, Supplementary Material online). SMC++ analysis showed a continual decline of all the domestic sheep populations during ∼8,000–10,000 years BP, which probably reflects the bottleneck effect of domestication (fig. 2*E*). Their subsequent population expansions show the expansion of agricultural in sedentary societies (Gignoux et al. 2011). Estimates of recent *N*_e_ by SNeP for the wild relatives and domestic sheep breeds ∼50 generations ago were ∼38–54 and 42–53, respectively. Relatively smaller *N*_e_ was observed in South-and-Southeast Asian and American domestic populations, which may be due to strong artificial selection and/or genetic isolation (Li et al. 2020).

### Phylogenetic Relationships and Population Structure

**Fig. 3.**
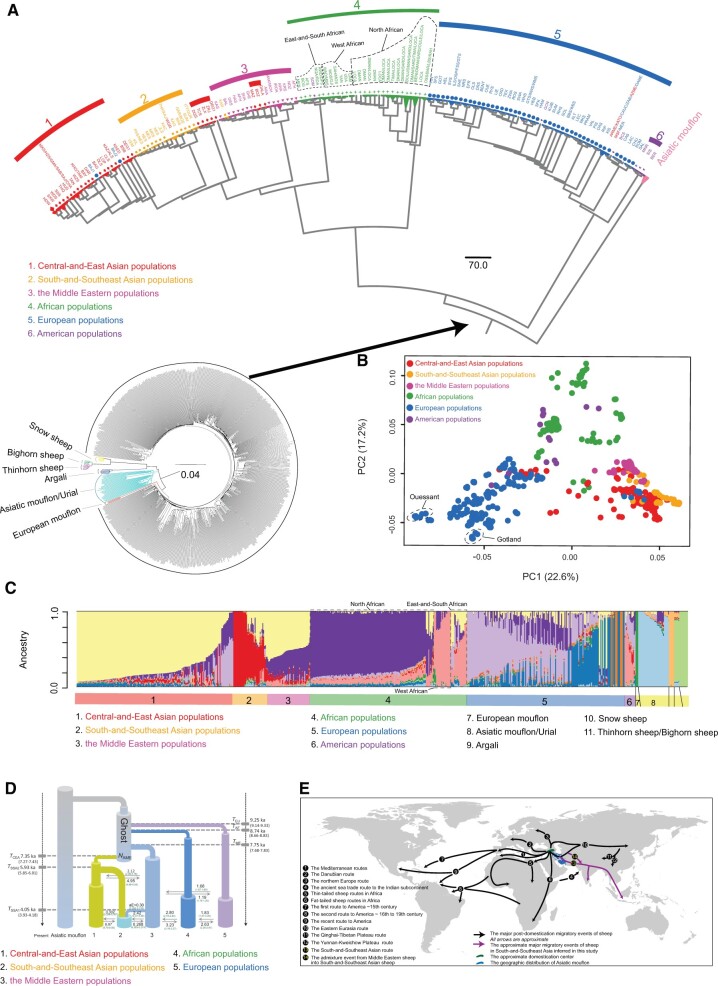
Population genetic structure of wild and domestic sheep and the demographic history. (*A*) Neighbor-joining (NJ) trees of wild and domestic sheep based on whole-genome SNPs using the identity-by-state (IBS) genetic distances implemented in PLINK v1.90 (Purcell et al. 2007). BAJ1 and BAJ2 are from the population BAJ, and DEG1 and DEG2 are from the population DEG. (*B*) PCA of domestic sheep populations. (*C*) Population genetic structure of wild and domestic sheep inferred from the sNMF analyses (*K* = 11) using whole-genome SNP data. (*D*) Demographic history reconstruction of domestic sheep. The best-supported demographic model of South-and-Southeast Asian population: South-and-Southeast sheep diverged from Central-and-East Asian population ∼5.93 ka and was later admixed by the Middle Eastern population ∼2.42 ka. The Middle Eastern population contributed ∼30% of their gene pool to the South-and-Southeast Asian population. Dates of events (ka) are indicated on the left and right, and estimated migration rates (migrants per generation) are scaled by the effective population size were given above/below the corresponding arrows. The 95% confidence intervals (CIs) are shown in parentheses. (*E*) Major sheep migrations across the world. (1) The Mediterranean routes (Ryder 1984), (2) the Danubian route (Ryder 1984), (3) the northern Europe route (Tapio et al. 2006), (4) the ancient sea trade route to the Indian subcontinent (Singh et al. 2013), (5) thin-tailed sheep routes in Africa (Muigai and Hanotte 2013), (6) fat-tailed sheep routes in Africa (Muigai and Hanotte 2013), (7) the first route to America ∼15th century (Dunmire 2013), (8) the second route to America ∼16th to 19th century (Spangler et al. 2017), (9) the recent route to America (Ryder 1984), (10) the Eastern Eurasia route (Lv et al. 2015), (11) the Qinghai-Tibet Plateau route (Zhao et al. 2017; Hu et al. 2019), (12) the Yunnan-Kweichow Plateau route (Zhao et al. 2017; Hu et al. 2019), (13) the South-and-Southeastern Asia route (this study), and (14) the admixture event from Middle Eastern sheep into South-and-Southeastern Asian sheep (this study).

Phylogenetic relationships among the wild and domestic sheep populations were inferred from whole-genome SNPs for 714 unrelated individuals (supplementary fig. S10 and table S13, Supplementary Material online). The phylogenetic tree (fig. 3*A*) shows a close relationship between the European mouflon and domestic sheep, followed by Asiatic mouflon. This supports the notion that the modern European mouflon is the feral descendent of early domestic sheep rather than a genuine species of wild sheep (fig. 3*A*) (Ciani et al. 2020). Domestic sheep were split into six geographically structured clades (Central-and-East Asian, South-and-Southeast Asian, the Middle Eastern, African, European, and American populations) with obvious explanations of apparent exceptions: the East European Baidarak sheep (blue) cluster within Central-and-East Asian sheep; thin-tailed Tibetan and Southwest Chinese sheep (red) are close to Indian breeds (yellow); Azerbaijan sheep (red) cluster within the Middle East sheep; and Asian fine-wool breeds (red) cluster are close to European Merino sheep (fig. 3*A*). The phylogenetic tree using the wild relatives as the outgroup revealed clear between-population genetic differentiation in domestic sheep as most of the samples from the same breeds clustered together. The Middle Eastern breeds were placed near the root within domestic sheep, being the descendant of the first domesticated sheep there (Zeder 2008). The European branch confirms separate subclusters for British and Nordic breeds, respectively (supplementary fig. S11, Supplementary Material online) (Kijas et al. 2012; Ciani et al. 2020), and the African branch indicates a genetic division between North-and-West African and East-and-South African breeds (Muigai and Hanotte 2013).

The geographic pattern is further supported by principal component analysis (PCA) and model-based clustering via the Admixture. A PCA plot of 653 unrelated domestic sheep shows largely separates the six geographic groups, most of which could be connected to continental origins (fig. 3*B* and supplementary fig. S11, Supplementary Material online). The inbred Ouessant and Gotland breeds have the extreme positions. European and African populations are largely separated from Asian populations with overlaps such as Merino-derived individuals (fig. 3*B*).

Genetic clustering analysis using sNMF (Frichot et al. 2014) recapitulates the same patterns observed in the phylogenetic tree (fig. 3*A*) and PCA (fig. 3*B*). At the optimal number *K* = 11 with the smallest cross validation error (supplementary fig. S12, Supplementary Material online), we observed five genetic clusters of the seven wild sheep species (Asiatic mouflon and urial are in the same genetic clusters; bighorn and thinhorn sheep are in one genetic cluster) and the six geographically distributed genomic components of domestic sheep (fig. 3*C*). Interestingly, sNMF analysis revealed the genetic components of wild relatives in domestic breeds, for example, the genetic ancestry of Asiatic mouflon in Awassi and Bangladeshi sheep breeds, argali in Bashibai sheep, and European mouflon in Cameroon and Svärdsjö sheep (fig. 3*C*). Additionally, we noted clear evidence of genetic heterozygosity among European, African, and Asian populations. This indicated substantial historical genetic flows among elite genetic stocks from different continents in the development of local breeds (Galal et al. 2008; Ciani et al. 2020).

One objective of this study was to fill the gaps of the global genomic coverage of population-scale WGS data by including samples from the so far neglected South-and-Southeast Asian, African, and South American sheep breeds. South-and-Southeast Asian breeds cluster with breeds from Pakistan and India. South American breeds share ancestry with sheep from both Western Africa and Europe as reported previously (Kijas et al. 2012).

### Genetic Origins of South-and-Southeast Asian Sheep

In order to explore the origin of South-and-Southeast Asian sheep (Singh et al. 2013; Lv et al. 2015), we tested three alternative models (supplementary fig. S13, Supplementary Material online). We computed the expected multidimensional site frequency spectra (SFS) under specific models and compared it with the observed multidimensional SFS by a composite likelihood method. The best supported SFS with the highest average value of estimated log_10_(likelihood) (supplementary fig. S14 and table S14, Supplementary Material online) suggested that South-and-Southeast Asian sheep descended from Central-and-East Asian population 5.85 to 6.01 ka (95% CIs). The optimal model also showed a later admixture from the Middle Eastern sheep 3.93–4.18 ka, which contributed approximately 29–32% of the gene pool (fig. 3*D* and supplementary table S15, Supplementary Material online).

The best model further indicated a divergence of the European, African, and the Middle Eastern sheep from their common ancestor 9.16–9.33, 8.66–8.83, and 7.68–7.83 ka, respectively (fig. 3*D* and supplementary table S15, Supplementary Material online). This was in agreement with the initial expansions of ancestral sheep as evidenced by the archaeological findings and early genetic studies (Zeder 2008; Deng et al. 2020). Asymmetric gene flow occurred from the Central-and-East Asia to the South-and-Southeast Asia (8.70–9.05 migrants per generation) and from the South-and-Southeast Asia to the Middle East (4.86–5.04 migrants per generation) (fig. 3*D* and *E* and supplementary table S16, Supplementary Material online).

### Retained or Introgressed Sequences from Wild Sheep in the Domestic Genomes

The sharing of ancestry by wild and domestic sheep, indicated by the Admixture analysis, may be the result of two scenarios. First, sequences from Asiatic and European mouflons that were inherited directly by the first domestic sheep may have been retained in specific breeds, whereas other parts of the genome adapted to the domestic status and breeding objectives, for instance during the transition from hair to wool sheep (Chessa et al. 2009). Second, introgression may have taken place during later contacts between wild and domestic sheep. Here we investigated the pattern and consequences of the retained or introgressed sequences (RIS) using a combination of *D* and *f*_d_ statistics.

**Fig. 4.**
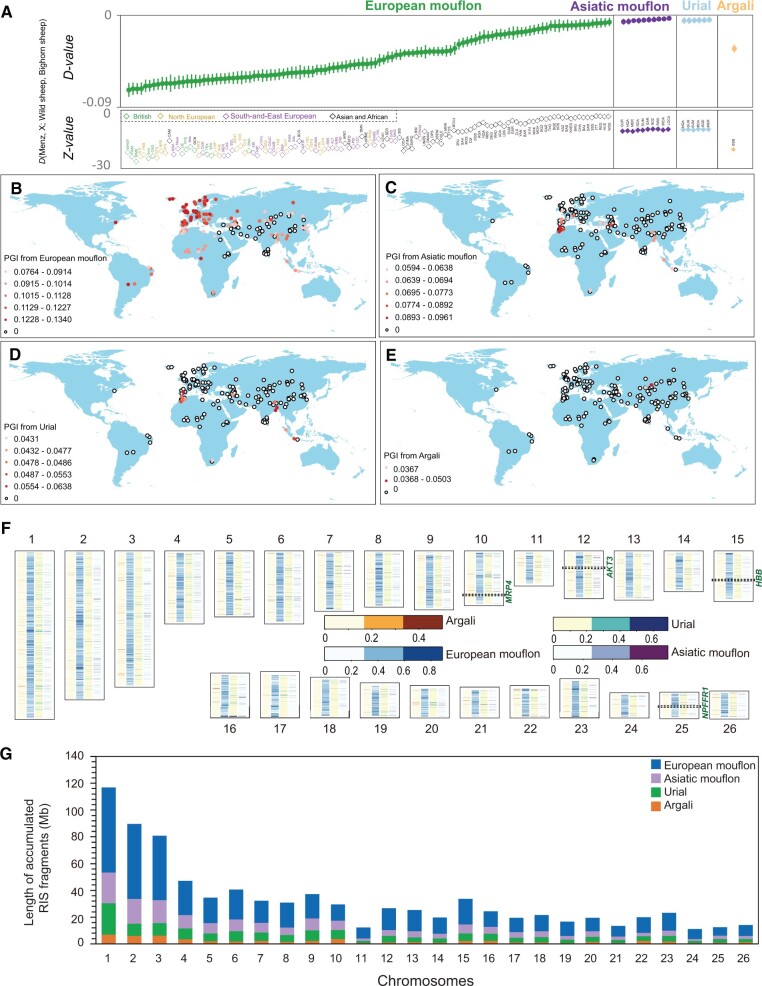
Genetic introgression of wild sheep species in domestic populations. (*A*) *D* statistic tests in the form (Menz sheep, X, wild sheep, Bighorn sheep) where wild sheep indicates European mouflon, Asiatic mouflon, urial, or argali, respectively. X indicate a candidate domestic population. Introgressions from European mouflon into British, North European, Southwest-and-East European, and Asian, and African populations are indicated by green, gold, purple, and black color, respectively. (*B*, *C*, *D*, *E*) The PGI across the whole genome of each wild sheep species in worldwide domestic populations. The levels of PGI from European mouflon, Asiatic mouflon, urial, and argali are mapped based on the results of *f*_d_ statistics: *f*_d_ (Menz sheep, X, European mouflon, Bighorn sheep), *f*_d_ (Menz sheep, X, Asiatic mouflon, Bighorn sheep), *f*_d_ (Menz sheep, X, Urial, Bighorn sheep), and *f*_d_ (Menz sheep, X, Argali, Bighorn sheep), separately. X indicates a candidate domestic population. PGI can be estimated with the equation: PGI =(Σ*f*_d__*i*_×*G_i_*)/*G*, where *f*_d__*i*_ and *G_i_* refer to the *f*_d_ value and the window size in base pairs for the *i*th window, and *G* is the genome size in base pairs (Zhou et al. 2020). (*F*) RIS of wild sheep species in domestic sheep on each chromosome. Colored heatmaps of blue, red, purple, and gray show the abundance of genomic regions containing RIS from the four wild sheep species based on *f*_d_ statistics (Argali, Asiatic mouflon, Urial, and European mouflon). The genes with the most significant signals of introgression are highlighted. (*G*) Length of accumulated RIS from the four wild sheep species (Argali, Asiatic mouflon, Urial, and European mouflon) in domestic sheep.

We used ABBA-BABA statistics to detect ancestry shared by wild donors to domestic breeds. In agreement with Ciani et al. (2020), we found substantial influence (negative *D* values) of European mouflon on several breeds with the higher |*Z*| values in North European sheep such as Welsh Mountain, Icelandic Leader, and Shetland (fig. 4*A* and supplementary table S17, Supplementary Material online). Retained or introgressed ancestry from Asiatic mouflon and the related urial are much lower (*D* value is closer to zero), although Z values indicate statistical significance for a few breeds. Asiatic mouflon and urial appear to have influenced the same breeds, confirming that these wild species have become mutually admixed (fig. 3*C*) (Demirci et al. 2013; Deng et al. 2020; Chen et al. 2021). Interestingly, the genomes of Bashibai sheep carry a sizable argali component. In addition to several other potential explanations such as horizontal gene transfer, hybridization, and common ancestry (Wang et al. 2021), the observation could be very likely the result of introgression (fig. 4*A* and supplementary table S17, Supplementary Material online).

To further locate the RIS genomic regions, we computed the *f*_d_ values for 100-kb windows and 50-kb step across the genomes of relevant domestic populations. After the *P*-values adjusted (false discovery rate, FDR), we detected 258 (*f*_d_ > 0.1714) ∼ 434 (*f*_d_ > 0.1585) significant introgressed blocks from argali, 283 (*f*_d_ > 0.2271) ∼ 335 (*f*_d_ > 0.2363) significant introgressed tracts from urail, 311 (*f*_d_ > 0.2374) ∼ 344 (*f*_d_ > 0.5322) significant introgressed blocks from Asiatic mouflon, 296 (*f*_d_ > 0.5117) ∼ 323 (*f*_d_ > 0.3157) significant introgressed tracts from European mouflon, respectively (supplementary fig. S15 and table S18, Supplementary Material online). The proportion of genome introgression (PGI) of domestic sheep breeds from different donor wild species on the basis of the *f*_d_ statistics gives different patterns (fig. 4*B*–*E* and supplementary table S19, Supplementary Material online). The proportion of RIS segments, which reflects the actual genome size of domestic sheep retained or introgressed from wild relatives was estimated to be 7.64–13.40%, 5.94–9.61%, 4.31–6.39%, and 3.67–5.03% of their whole genomes originating from European mouflon, Asiatic mouflon, urial, and argali, respectively (supplementary table S19, Supplementary Material online). Counting the RIS tracts of union set for every wild donor into all domestic recipients yielded 566, 1437, 1718, and 4808 RIS tracts from argali, urial, Asiatic mouflon, and European mouflon, respectively (fig. 4*F* and supplementary table S20, Supplementary Material online). The accumulated genome sizes of RIS segments on each chromosome in domestic genomes were shown in figure 4*G*. The number and size of detected introgression windows show different patterns among different chromosomes and different donor wild species (fig. 4*F* and *G* and supplementary table S20, Supplementary Material online).

Of the tracts shared with the wild relatives, 79 common blocks were shared with all four wild species and span 125 functional genes (supplementary tables S21 and S22, Supplementary Material online). In the GO analysis of the retained or introgressed genes, we identified a significant (adjusted *P* < 0.05) overrepresentation of genes involved in organonitrogen compound (GO:0010243), hemoglobin complex (GO:0005833), olfactory receptor activity (GO:0004984), and oxygen transporter activity (GO:0005344) (supplementary fig. S16 and table S23, Supplementary Material online). The KEGG analysis identified a major and significant enrichment of genes associated with insulin signaling pathway and African trypanosomiasis (oas05143) (supplementary fig. S16, Supplementary Material online). In particular, the significantly enriched GO terms and KEGG pathways contained the oxygen transporter activity, patterning of blood vessels, and olfactory receptor activity genes such as *HBB*, *VEGFC*, and *Olr51L1*.

**Fig. 5.**
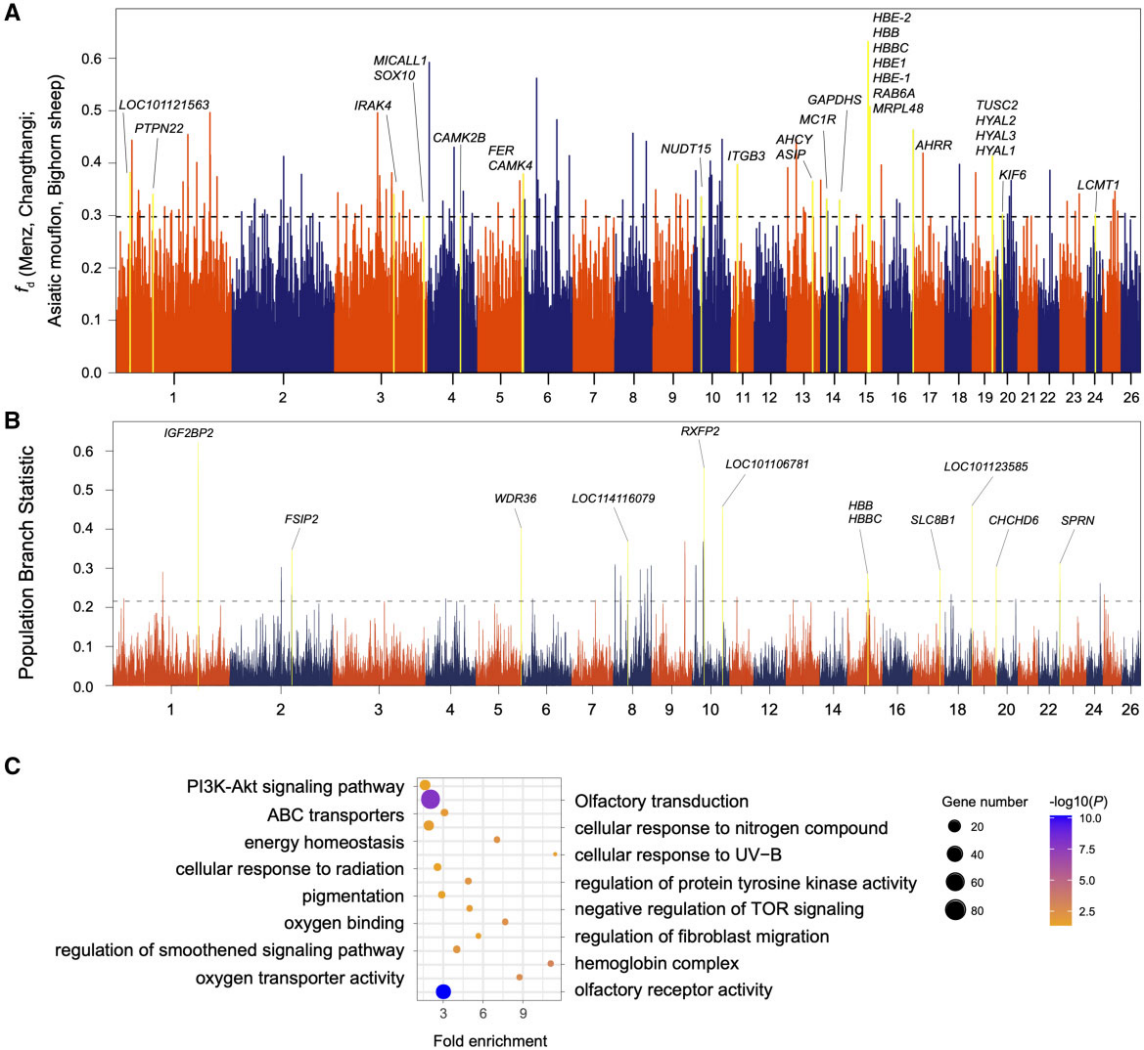
Signals of introgression and selection in the Changthangi sheep. (*A*) Manhattan plot showing the introgression signal from Asiatic mouflon to Changthangi sheep. The locations of the *HBB*, *HBE2*, *HBBC*, *HBE1*, *RAB6A*, and *MRPL48* genes are shown by the highest *f*_d_ value. (*B*) Signals of selection in the Changthangi sheep detected by the PBS (Yi et al. 2010). PBS values in windows of 100 kb and names of genes associated with the highest peaks are shown. (*C*) GO and KEGG pathway enrichment analysis based on genes across significant introgressed regions from wild species to Changthangi sheep. The dot size shows the gene count enriched in the pathway, and the dot color shows adjusted significance value of the enriched pathways.

**Fig. 6.**
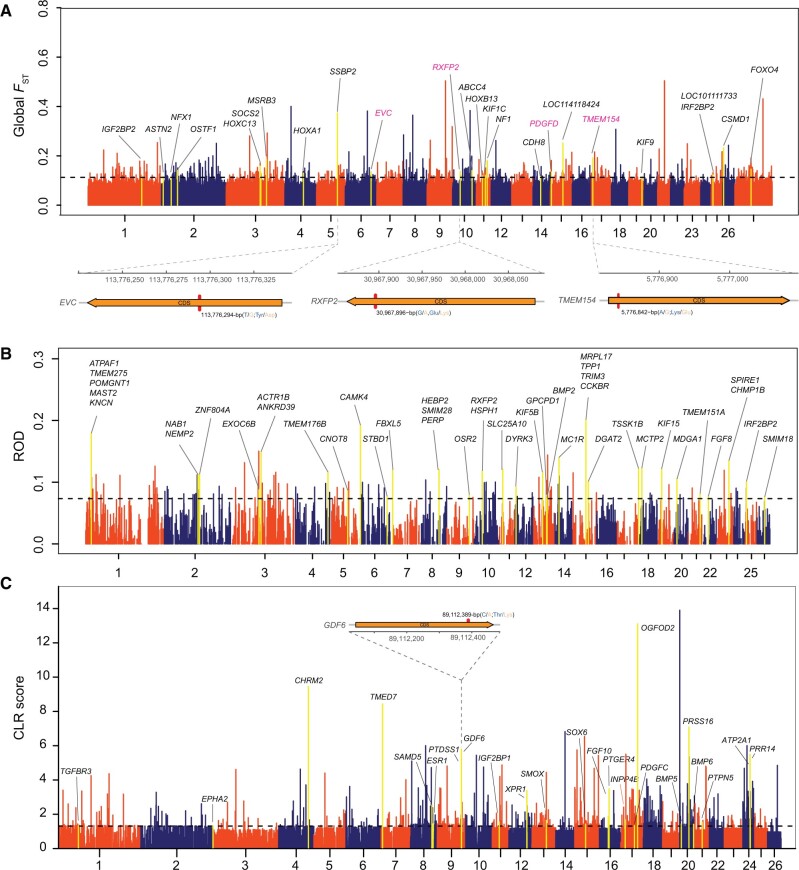
Genome-wide selective signals across all the domestic sheep populations based on global *F*_ST_ and ROD, and associated with the stature (i.e., body size) phenotype. (*A*) Genome-wide distribution of global *F*_ST_ across all the 158 domestic sheep populations. (*B*) Candidate selective genomic regions based on ROD analysis. (*C*) Manhattan plot for the dwarf (i.e., small body size) phenotype showing the CLR score distribution in the Ouessant sheep population. Genes within regions with significant outlier scores are highlighted.

In order to illustrate the potential of this approach, we focus on the Changthangi sheep in the high-altitude region of Ladakh *ca.* 4,000 m above sea level. Genome scans of *f*_d_ values of Changthangi versus Asiatic mouflon were shown in figure 5*A*. One of the introgression signals is the *HBB* region on chromosome 15 (chr15: 51.9–52.0 Mb). Various analyses such as the population branch statistics (PBS, Yi et al. 2010; fig. 5*B* and supplementary table S24, Supplementary Material online), *d*_xy_, *F*_ST_ distances, and haplotype pattern (supplementary fig. S17, Supplementary Material online) suggested an anomalous phylogeny as the result of wild introgression of variants of *HBB* and/or neighboring genes that confer hypoxia adaptation in the Changthangi sheep (Hu et al. 2019). In the GO analysis of the introgressed genes from wild species into Changtangi sheep, we identified a significant (*P* < 0.05) overrepresentation of genes involved in oxygen transporter activity (GO:0005344), oxygen binding (GO:0019825), pigmentation (GO:0043473), cellular response to UV-B (GO:0071493), and energy homeostasis (GO:0097009) (fig. 5*C*). The KEGG analysis identified a major and significant enrichment of genes associated with olfactory transduction (oas04740) and PI3K-Akt signaling pathway (oas04151) (fig. 5*C*). The two highest peaks in the PBS scan (fig. 5*B*) cover *IGF2BP2*, encoding an important protein in cell metabolism and development, and *RXFP2*, a gene involved in horn development (Li et al. 2018; Aldersey et al. 2020). Both also have elevated *F*_ST_ values (fig. 6*A*).

We calculated the probability of incomplete lineage sorting (ILS) using a recombination rate of 1.5 cM/Mb (Petit et al. 2017), a generation time of 3 years for domestic sheep (Zhao et al. 2017), a divergence time of 2.36 Ma between argali and domestic sheep (Yang et al. 2016), ∼1.26 Ma between urial and domestic sheep (Rezaei et al. 2010), ∼11 ka between Asiatic mouflon and domestic sheep (Chessa et al. 2009), and 5–6 ka between European mouflon and domestic sheep (Vigne 1992). This gives a length of tracts in domestic sheep shared with argali of 42.4 bp, with urial of 79.4 bp, with Asiatic mouflons of 9,091 bp, and with European mouflon of 16.7–20 kb. The probability of a length of 85.4 kb (i.e., the observed introgressed region from argali, urial, Asiatic mouflon, and European mouflon) is zero, which ruled out that the introgressed region identified in Changthangi sheep were due to random ILS without selection.

### Novel Signatures of Selection

The distribution of genetic differentiation statistic *F*_ST_ values for 50-kb windows with 25-kb step across the genomes of domestic sheep, an indicator of selection, is summarized in figure 6*A* and supplementary table S25, Supplementary Material online. Selective sweeps contained 559 genes, of which 259 (46%) were novel, and 300 were identified by several previous investigations on the basis of WGS (supplementary table S25, Supplementary Material online) (Yang et al. 2016; Alberto et al. 2018; Li et al. 2020). Functional annotation of putatively selected genes revealed that they were predominantly associated with morphological and agronomic traits, including genes such as *KRT71* and *FGF7* (skin development function), *Olr226*, *Olr51B2*, and *Olr2D3-like* (olfactory function), and *HBB*, *HBB-like*, *HBBC*, and *HBE2* (oxygen transporter function, see above). In addition, the previously characterized genes with relevant traits such as *RXFP2* (see also fig. 5*B*), *PDGFD* (Dong et al. 2020), and *TMEM154* (Heaton et al. 2012; Hu et al. 2019; Li et al. 2020) have elevated *F*_ST_ values (fig. 6*A*).

We found significant overlap of the *F*_ST_ outliers with known QTLs in sheep (permutation test, *P* < 0.001), most notably with milk-, meat-, and disease-resistance-related QTLs (supplementary table S26, Supplementary Material online). Within the protein-coding exons in the selective regions, we identified 8,753 nonsynonymous SNP mutations in 559 putatively selected genes. The allele frequencies of nonsynonymous SNPs and their genotypes pattern in ten genes (e.g., *EVC*, *GDF6*, *RXFP2*, *TMEM154*) showed differences among populations with varied phenotypes such as body size (e.g., normal/dwarf), horn status (e.g., horned/polled), and disease resistance (e.g., pneumonia susceptibility/resistance) (supplementary table S27, Supplementary Material online).

A genome-wide reduction of diversity (ROD) as alternative selection signature indicated 134 genomic regions with top 1% of both ROD values between landraces and improved breeds of domestic sheep (fig. 6*B* and supplementary table S28, Supplementary Material online). We found that ROD chr15: 49,000,001–50,000,000 were in concordance with high nonsynonymous to synonymous (N/S) ratio (N/S ratio = 1.49; supplementary table S29, Supplementary Material online). Additionally, 59 SVs under selection during sheep improvement including 1,601 candidate genes (supplementary table S30, Supplementary Material online), several of which are related to regulation of ovulation rate (e.g., *BMP15* and *CYP17*) (Estienne et al. 2017).

The composite likelihood ratio (CLR) statistic scores for 10-kb windows in Ouessant sheep with an extreme dwarf phenotype was shown in figure 6*C*. Outlier windows with CLR > 1.31 (*P* < 0.001) were considered to be under artificial selection and cover 1,702 genes (supplementary table S31, Supplementary Material online). These genes were mainly involved in bone resorption (e.g., *PTGER4*), bone mineralization (e.g., *BMP6*), and dwarfism (e.g., *GDF6* and *PTDSS1*). In gene *GDF6*, we also detected significant (Mann–Whitney, *P* < 0.001) differences in a few nonsynonymous SNP variants and genotype patterns between the populations with different statures (supplementary fig. S18, Supplementary Material online). Several of the genes (e.g., *IGF2BP1, SMOX family member*, and *FGFR family member*) related to stature in sheep have been associated with body size in cattle and dogs (Rimbault et al. 2013; Hayward et al. 2016; Bouwman et al. 2018).

### Selective Signatures Associated with Wool Traits

**Fig. 7.**
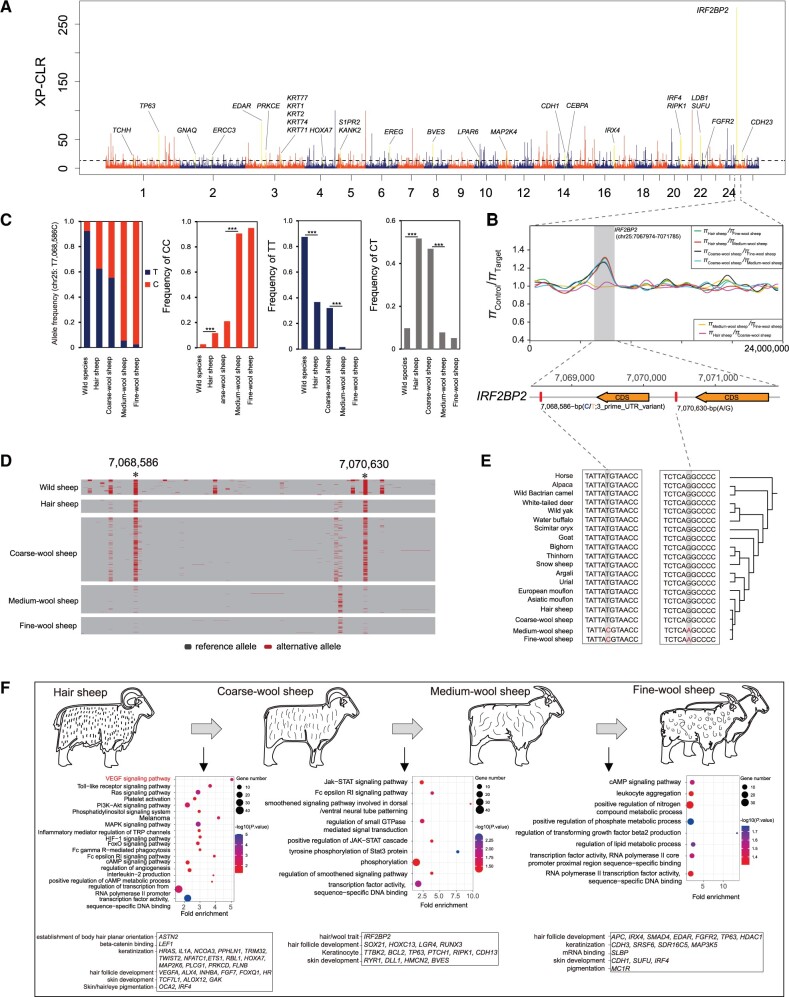
Genomic evolution of hair/coarse-wool/medium-wool/fine-wool sheep. (*A*) Whole-genome selective signals between fine-wool and hairy populations of domestic sheep by the cross-population composite likelihood ratio (XP-CLR) test. (*B*) Six comparisons of π ratio (π_Hair sheep_/π_Coarse-wool sheep_, π_Hair sheep_/π_Medium-wool sheep_, π_Hair sheep_/π_Fine-wool sheep_, π_Coarse-wool sheep_/π_Medium-wool sheep_, π_Coarse-wool sheep_/π_Fine-wool sheep_ and π_Medium-wool sheep_/π_Fine-wool sheep_) indicates strong selective signal in *IRF2BP2* gene. (*C*) Allele frequency of the mutation site and frequencies of genotypes CC, TT, and CT in 3′-UTR of *IRF2BP2* (chr25: T7,068,586C), and significance of the genotype difference is tested. (*D*) Haplotype pattern of *IRF2BP2* among wild sheep, hairy, coarse-wool, medium-wool, and fine-wool populations of domestic sheep. (*E*) Sequence comparison among different species at mutations chr25: 7,068,586, and chr25: 7,070,630. (*F*) GO and KEGG pathway enrichment analyses, with the significant (*P* < 0.05) GO terms and pathways and associated genes shown.

Genome-wide XP-CLR selection tests of domestic breeds possessing different fleece fiber variations (e.g., hair, coarse wool, medium wool, and fine wool; supplementary table S32, Supplementary Material online) were shown in all the six pairwise comparisons (fig. 7*A* and supplementary figs. S19–S23 and tables S33–S38, Supplementary Material online). From 2,759 genes associated with 5,995 selective signals, 86 are scored in at least two of the six comparisons and with functions involved in fiber and wool development, including novel (e.g., *HR*, *ASTN2*, *TWIST2*, *IRF4*, *INHBA*, and *FGF7*) and previously reported (e.g., *MCIR*, *FOXQ1*, *TP63*, *VEGFA*, and *PTCH1*) genes. We also identified 414 selective SVs associated with wool traits in the selective sweeps of SVs (supplementary tables S39–S44, Supplementary Material online). Annotation of the SVs indicated their colocalizations with genes or QTLs associated with mean fiber diameter (e.g., *FST* gene) (Ma et al. 2017). Positional gene enrichment analysis of the gene sets showed significant (*P*_adj._ < 0.05) overrepresentation of genes related to fiber diameters in these regions (De Preter et al. 2008). All candidate genes under selection are directly or indirectly associated with wool growth and development (fig. 7*F*).

We further dissected the genomic architecture of these strong candidate genes by calculating allele frequencies at nonsynonymous SNPs in exons. We observed significant differences in allele frequencies (Mann–Whiteney test, *P* < 0.001), and genotype or haplotype patterns of nonsynonymous SNPs in *IRF2BP2* and *MC1R* genes between breeds displaying different wool phenotypes (supplementary table S27 and fig. S24, Supplementary Material online).

### Functional Analysis of *IRF2BP2* Variation

Remarkably, the signals overlapping with *IRF2BP2* are outliers in four of the six comparisons in the XP-CLR analysis and *π* ratio analysis (fig. 7*B* and supplementary tables S26–S31, Supplementary Material online). We annotated the gene containing 85 SNPs and detected one intron and two exons in the gene. The 3′-UTR variant (chr25: 7,068,586) and the intronic mutation (chr25: 7,070,630) showed significantly (*P* < 0.05) differentiated allele and genotype frequencies between the groups of breeds with different fleece fiber diameters, such as hair, coarse wool, medium wool, and fine wool (fig. 7*C* and supplementary fig. S25, Supplementary Material online). In domestic sheep, the ancestral *T* (chr25: 7,068,586) and *G* alleles (chr25: 7,070,630) showed higher frequencies in hair and coarse-wool populations, corresponding to the ancestral alleles, whereas the derived reference *C* (chr25: 7,068,586) and *A* (chr25: 7,070,630) alleles were dominant in medium-wool and fine-wool populations (fig. 7*C* and *D*). Further characterization of alleles of these two variants in a large variety of species including wild sheep, wild yak, goat, and horse showed both ancestral alleles in animals with hair or coarse-wool coat (fig. 7*E*). Thus, this observation indicated that the two variants in *IRF2BP2* may have advantageous effects on fleece fiber diameter. In our worldwide sheep panel, these SNPs do not cosegregate with the *EIF2S2* insertion upstream of the *IRF2BP2* gene previously found to be associated with wool and fine-wool genotype (Demars et al. 2017).

**Fig. 8.**
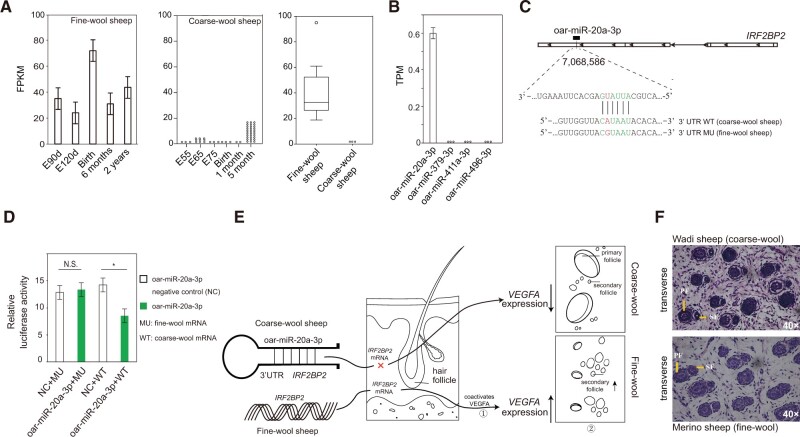
Histological analysis, mRNA, and miRNA expressions and genetic mechanisms for the gene *IRF2BP2* and oar-miR-20a-3p involved in the fleece fiber variation in domestic sheep. (*A*) Expressions of *IRF2BP2* in embryonic developmental stages and 2 years old of fine-wool (left), coarse-wool sheep (medium) and boxplot of FPKM distribution between fine-wool and coarse-wool sheep (right). (*B*) Expressions of miRNAs in coarse-wool sheep (Hu sheep) normalized by reads per million reads mapped to miRNAs (RPM). (*C*) Putative binding sites for oar-miR-20a-3p to the 3′-UTR of *IRF2BP2*. (*D*) Dual-luciferase reporter assays, *P < 0.05 (*t*-test); N.S., not significant. (*E*) Proposed genetic mechanisms for *IRF2BP2* and oar-miR-20a-3p involved in the fleece fiber variation in domestic sheep. ①Ho et al. (2012) and ②Zhang et al. (2018). (*F*) Histological transverse section of skin in Chinese Merino (fine wool, at embryonic 85 days) and Wadi sheep (coarse wool, at embryonic 90 days); PF, primary wool follicle; SF, secondary wool follicle.

The fragments per kilobase million (FPKM) value for *IRF2BP2* mRNA expression showed significant (*P* < 0.05) difference in skin tissues at different prenatal stages between coarse-wool and fine-wool sheep (fig. 8*A* and supplementary table S45, Supplementary Material online). No expression was observed in *IRF2BP2* for coarse-wool sheep (fig. 8*A*). We identified binding sites of the highly conserved miRNAs *oar-miR-496-3p*, *oar-miR-379-3p*, *oar-miR-411a-3p*, and *oar-miR-20a-3p* binding in the 3′-UTR of *IRF2BP2*, which suggested *IRF2BP2* as a target of these four miRNAs (supplementary table S46, Supplementary Material online). MiRNA expression profiling in skin samples of three coarse-wool Hu sheep and nine Aohan fine-wool sheep (supplementary table S47, Supplementary Material online) revealed expression of only *oar-miR-20a-3p* in coarse-wool sheep (fig. 8*B*).

Binding of *oar-miR-20a-3p* to the 3′-UTR of wild-type (WT) *IRF2BP2* (fig. 8*C*) was confirmed by luciferase assay in HEK293T cells. We introduced the WT *IRF2BP2* 3′-UTR into a luciferase expression vector and found that expression of *oar-miR-20a-3p* reduced reporter activity compared with a negative control mimic (fig. 8*D*). However, mutated 3′-UTR of *IRF2BP2* abolished the reduction of luciferase by the *oar-miR-20a-3p*-mediated (fig. 8*D* and *E* and supplementary fig. S26 and table S46, Supplementary Material online). Altogether, these data supported that the WT *IRF2BP2* 3′-UTR was a direct and functional target of *oar-miR-20a-3p*, which could directly bind *IRF2BP2* and potentially regulate *IRF2BP2* expression in the skin tissues of coarse-wool sheep.

We also detected a strong and consistent selective signal in the gene *VEGFA* (vascular endothelial growth factor A) in four comparison tests (hair vs. fine wool, hair vs. medium wool, coarse wool vs. fine wool, and coarse wool vs. medium wool, supplementary tables S26–S31, Supplementary Material online). Early evidence suggested that *IRF2BP2* coactivates *VEGFA* expression, leading to the activation of secondary wool follicles (Ho et al. 2012; Li, Lu, et al. 2012; Li, Man, et al. 2012; Zhang et al. 2018). In the histological analysis, hematoxylin and eosin (H&E) staining showed a larger ratio of diameters (*D*_S_/*D*_P_) but a smaller number (*N*_S_/*N*_P_) of secondary to primary wool follicles in Wadi sheep (coarse wool) than in Chinese Merino sheep (fine wool) in the important developmental stage of wool follicles (fig. 8*F*), which consequently accounted for the higher yields and finer wool of uniformly small-diameter fibers in fine-wool sheep (Galbraith 2010). Thus, *oar-miR-20a-3p* may act on the development of wool follicles in coarse-wool sheep through its effect on *IRF2BP2*, which potentially inhibits *VEGFA* signaling expression in wool follicles (fig. 8*F*).

## Discussion

In this study, we performed a whole-genome sequencing analysis of 810 wild and domestic sheep. This is thus the hitherto most comprehensive data set on population genetic structure of worldwide sheep using more than ∼2 times as many individuals and WGS as in previous studies (Alberto et al. 2018; Naval-Sanchez et al. 2018; Deng et al. 2020; Li et al. 2020). We explored the occurrence of sequences from wild species in the domestic genomes and further identified useful nonsynonymous SNP mutations and SVs involved local adaptation and agronomic traits.

### The Influence of Wild Species on Domestic Genomes

In general, a high level of diversity with novel, rare, and private variants was observed in wild relatives compared with both improved and landrace breeds (fig. 1*C* and *D*). In domestic sheep, we observed many region-specific variants and a clear clustering of populations according to their geographic origins (supplementary fig. S3, Supplementary Material online). Several valuable genetic variants for modern sheep appeared to originate from wild sheep species. Genetic influence of wild on domestic species as a mode of adaptation have already been described for chicken (Wang et al. 2020), pigs (Ai et al. 2015), dogs (Miao et al. 2016), and cattle (Chen et al. 2018) and is supported by ancient DNA studies (Frantz et al. 2020). We observed that among the wild sheep species, the European mouflon has the most widespread influence on domestic sheep, although the influence of Asiatic mouflon may have been underestimated by a substantial influence of the urial on the current Asiatic mouflon population (this study) (Demirci et al. 2013; Deng et al. 2020). The European mouflon is supposed to have emerged as a feral population when the original hair-coat domestic sheep were replaced by wool sheep. During this process, mouflon DNA may have been retained in domestic sheep, in particular in North European breeds, some of which have retained a wild appearance (Chessa et al. 2009; Ciani et al. 2020; Deng et al. 2020). Several English breeds such as the Suffolk, Texel, Dorset, and the Longwools became transboundary mutton sheep and have been used for improving local breeds on different continents (Kijas et al. 2012; Deniskova et al. 2018; Cao et al. 2021), thus spreading the ancestry of European mouflon.

Alternatively, mouflon influence may be the result of a later introgression in a sympatric domestic population (Barbato et al. 2017; Cao et al. 2021). Historical records from ancient Rome and the 18th century have described hybridization between wild and domestic sheep in Europe (Cetti 2000). Even nowadays, farmers in Sardinia typically allow sheep to graze in the wild, where European mouflon reside (Lorenzini et al. 2011). Our estimates of the proportion of the mouflon component in domestic breeds (7.64–13.40%) are higher than the 1.0–4.1% estimated by Barbato et al. (2017) and the 0.6–1.3% estimated by Cao et al. (2021), both of which were on the basis of 50K genotypes. This discrepancy is likely explained by sensitive detection of short mouflon fragments by WGS, which are missed by 50K SNP BeadChip genotyping with an average spacing between SNPs of about 54 kb. Introgression is the most plausible explanation for the argali influence on the Bashibai sheep. We observed that domestic sheep may have acquired advantageous alleles of several immune and sensory genes via natural or managed hybridization with the wild relatives (supplementary table S22, Supplementary Material online). This may very well have facilitated the worldwide range of domestic sheep adapting to a wide range of environments (Cao et al. 2021), for instance to the extreme conditions of the Arctic climate and the Himalaya Highland.

Previous studies have showed genetic introgression of wild relatives in domestic animals such as sheep, goat, pig, dog, cattle, yak, cattle, rabbit, horse, and chicken (Ai et al. 2015; Medugorac et al. 2017; Jones et al. 2018; Wu et al. 2018; Lawal et al. 2020; Wang et al. 2020; Zheng et al. 2020; Cao et al. 2021; Yu et al. 2021; ). The proportion of wild introgression varies from 0.05% to 27.3% depending on the populations of domestic animals and the relevant wild relatives (supplementary table S48, Supplementary Material online). These introgressed variants and segments were located in genes with functions such as environmental adaptation (e.g., the response to hypoxia), innate immunity, olfactory relevance, and morphological traits (e.g., coat color, the polled, and the different shapes of horns). Introgressed variants in genes associated with similar functions such as the oxygen transportation and hemoglobin complex have been observed in this and early studies (Hu et al. 2019). Nevertheless, genes with different functions were also detected in these studies (supplementary table S48, Supplementary Material online), which could be due to differentiated natural and artificial selection forces in a diverse range of environments and varied production systems.

### Demographic History of South-and-Southeast Asian Sheep

Reconstruction of the history of the Asian sheep populations by demographic modeling suggested that Central-and-Eastern and South-and-Southeast Asian sheep diverged 5.85–6.01 ka with subsequent gene flow from the Middle East 3.93–4.01 ka. This was possibly mediated by gene flow along the Southern Silk Road route and the Tibet–Nepal and Burma–India routes (Pe 1959; Shaha 1970; Lv et al. 2015). In the Holocene and Neolithic time, the major transformation of farming and pastoralism in Eurasia were accompanied by human migrations (Narasimhan et al. 2019) and cultural transitions, such as the Late Neolithic to Early Bronze Age material cultures in the Near East, the Kura-Araxes culture in the Southern Caucasus, the Yamnaya culture across the Eurasian Steppe, and the Indus Valley Civilization (Narasimhan et al. 2019; Skourtanioti et al. 2020). Our findings are consistent with recent paleogenomic evidence for the dynamic population history on the Eurasian Steppe and the genetic makeup of most present-day South-and-Southeast Asian populations during the Early and Late Bronze ages, which share ancestry of: 1) Anatolian and Iranian farmers across the broad region of Iranian plateau ∼6,500–6,000 BP (Narasimhan et al. 2019; Raghavan et al. 2019; Shinde et al. 2019); and 2) North Eurasian and Central Asian Steppe pastoralists along the Inner Asian Mountain Corridor, the ancient “Silk Roads” across Asia (e.g., Kazakhstan, Turkmenistan, Afghanistan, and Pakistan) ∼4,500–4,000 BP (Majumder 2010; Frachetti et al. 2017; Lipson et al. 2017; Narasimhan et al. 2019). Thus, our results illustrate the impact of the Middle Eastern farming to both the sheep husbandry in Iran and to the pastoralism on the Eurasian Steppe spreading eastward into South-and-Southeast Asia (Lazaridis et al. 2016).

The demographic reconstruction and population genetic differentiation supported close genetic relationship between South-and-Southeastern Asian and Central-and-Eastern Asian populations (fig. 3*A*–*D*). Our results supported a relatively recent divergence and a close genetic affinity between South-and-Southeastern Asian and Chinese sheep populations (including Tibetan and Southwestern Chinese populations). The observation could also be explained by the strong gene flow from Central-and-Eastern Asian populations to South-and-Southeastern Asian populations indicated by the modeling analyses (fig. 3*D*), which could be through several popular ancient land (the Southern Silk Road route and the Tibet–Nepal and Burma–India routes) (Pe 1959; Shaha 1970; Lv et al. 2015). The later Near Eastern infusion could be associated with the introduction of fat-tailed sheep, which originated in the Near East (around 5,000 years BP; Ryder 1984), as supported by the Y-chromosomal genetic variation analyses (Deng et al. 2020). Alternatively, an early mtDNA study suggested that sheep could have been introduced to Indian subcontinent from Near East, probably by ancient sea trade route (Singh et al. 2013). Overall, our study complemented the origins and migrations of sheep in previously understudied regions and presented a comprehensive picture of expansions of sheep across the world (fig. 3*E*). We noted that the estimated time of the main demographic events were in the approximate periods of the recent paleogenomic studies (Lazaridis et al. 2016; Frachetti et al. 2017; McColl et al. 2018; Narasimhan et al. 2019; Shinde et al. 2019; Skourtanioti et al. 2020), but the time difference observed here could be due to a limited number of modern samples and the lack of ancient samples in Central, South-and-Southeast Asia. Also, there were controversial estimates of time for the population demographic histories in these paleogenomic investigations. Additional samples in the regions (e.g., Kazakhstan, Afghanistan, Pakistan, Vietnam, Thailand, and Malaysia) including particularly ancient DNA will be needed to elucidate the genetic origins and prehistoric population interactions of sheep in South-and-Southeast Asia. Our results of sheep history in South-and-Southeast Asia can provide insights into the waves of human migration in the regions.

For a more complete visualization of the major migrations of domestic sheep (fig. 3*E*), we combined the evidence of the modeling for the Asian populations (fig. 3*D*) with 1) the topology with separate positions for British and Nordic breeds (fig. 3*A* and supplementary fig. S11, Supplementary Material online) (Tapio et al. 2006; Kijas et al. 2012; Ciani et al. 2020); 2) the clear separation of North-and-West African sheep and East-and-South African sheep (fig. 3*A* and supplementary fig. S11, Supplementary Material online); and 3) tentatively, the model-based clustering for the American populations (fig. 3*C*) (Kijas et al. 2012), which is in agreement with earlier results based on mtDNA and SNP BeadChip (Spangler et al. 2017; Paim et al. 2021).

### Molecular Basis of Adaptive and Agronomic Traits

We performed several alternative methods for genome-wide searching of selection signatures in order to identify novel trait-associated genes and variants. With our data set, PBS, *F*_ST_, and CLR gave clear signals above a uniform background, which is in contrast to rather noisy *f*_d_ and ROD scans. In general, the various approaches each detect different panels of genes with only little overlap, indicating for all methods a high frequency of false negatives. At the same time, all methods are prone to delivering false positives, further complicating the interpretation (Utsunomiya et al. 2015; Saravanan et al. 2020). On the other hand, our large WGS data set allows to identify candidate causative mutations, at least in coding sequences but also in plausible miRNA binding sites, and to test their correlation with the breed-dependent phenotypes. In addition, alignment of haplotype patterns (figs. 5*D* and 7*D*) for selected regions appeared to be informative.

From the multitude of potentially informative selection signatures, we have highlighted *HBB* and neighboring genes (adaptation to hypoxia), *EVC* (development), *RXFP2* (horn development), *TMEM154* (disease resistance), *GDF6* (growth), *PDGFD* (tail configuration), and *MC1R* (coat color), all of which are key genes for the most relevant phenotypes of domestic sheep. For fleece fiber variation, we found an association of mutated allele chr25:7,068,586(C) in the 3′-UTR of *IRF2BP2* with the medium- or fine-wool phenotype. Although we observed homozygous genotypes of ancestral and derived alleles at the SNP mutation (chr25: T7,068,586C) in the hair and coarse-wool sheep populations, the ancestral allele chr25:7,068,586(T) showed much higher frequencies than those in medium-wool and fine-wool sheep (fig. 7C). Thus, we concluded the ancestral allele chr25:7,068,586 (T) is associated with the phenotypes of hair and coarse wool in wild and domestic sheep.

Variation between hairy and woolly fleece has been previously found to be associated with an insertion of an antisense *EIF2S2* retrogene in the 3′-UTR of *IRF2BP2*, using a mainly French panel of populations (Demars et al. 2017). However, this association could not be entirely reproduced in our worldwide panel of wild and domestic sheep, particularly in the wild species (with the phenotype of hair), hair sheep, and coarse-wool sheep populations (supplementary fig. S27, Supplementary Material online). We estimated LD between the mutation (chr25: T7,068,586C) and the insertion (as*EIF2S2*) by calculating the parameter of LD (*r*^2^) (Hill and Robertson^1968^). We found strong linkage between them in wild species (frequency of *IRF2BP2^wt^* = 1, frequency of *T* = 0.96) and fine-wool (*r*^2^ = 0.27) sheep populations, whereas lower (*r*^2^ < 0.1) or no linkage was observed in the hair, coarse-wool, and medium-wool sheep populations (supplementary table S49, Supplementary Material online). We noted that only seven fine-wool individuals were included in the LD estimation, which might have inadequate statistical power and significance. The results showed cosegregation between the ancestral allele chr25:7,068,586T and the absence of *EIF2S2* insertion in the 3′-UTR of *IRF2BP2* in wild sheep species. Thus, the expression of *IRF2BP2* was regulated by both the *EIF2S2* insertion and the SNP (chr25: T7,068,586C) in the 3′-UTR, which is associated with the fleece fiber phenotypes in wild and domestic sheep. Our results provided complementary evidence for the genetic mechanisms underlying the fleece fiber variation in sheep. Different from Demars *et al.* (2017), we did not find *IRF2BP2^asEIF2S2^*/*IRF2BP2^asEIF2S2^* homozygotes. The difference in genotype pattern between this and the previous study could be due to that 1) multiple approaches and strict thresholds have been adopted in obtaining the accurate genotypes; 2) the *EIF2S2* insertion was genotyped in much less samples of medium- and fine-wool sheep here, which, however, showed a high frequency in medium- and fine-wool sheep (Demars *et al.* 2017).

Besides these, we detected a set of candidate genes associated with fleece fiber variation in sheep. Among these and previously identified candidate genes, 7 genes (e.g., *VEGFA*, *HRAS*, and *AKT*) were located in the pathways of VEGF signaling, which plays a central role in regulating cell proliferation, migration, and survival; 17 genes (e.g., *CACNA2D3*, *FGF7*, and *MAP4K4*) were found in MAPK signaling pathway, and 16 genes (e.g., *EFNA5*, *PIK3R3*, and *VTN*) were involved in the PI3K-Akt signaling pathway, which were functionally related to cell proliferation and survival (supplementary fig. S28, Supplementary Material online). Functions of these genes and network of these relevant pathways indicated that *IRF2BP2*, *VEGFA*, and *oar-miR-20a-3p* are the potentially important factors regulating the fleece fiber diameter in domestic sheep.

We conclude that the historical wild-relative genetic introgression played an important role in shaping the genetic diversity and agronomic traits and introduced adaptive variants in domestic sheep. The large data set presented here is a useful resource by providing a genomic framework for exploration of the genomic basis underlying phenotypic traits and genetic improvement of sheep.

## Materials and Methods

### Data Sets, Samples, and DNA Extraction

The sequencing data sets consisted of WGS generated in this study and published previously, totaling 810 individuals from 7 wild relatives (72 individuals) and 158 domestic sheep populations (738 individuals) around the world. The novel sequences included genomes of one Asiatic mouflon and 165 individuals from 82 domestic populations (fig. 1*A* and supplementary table S1, Supplementary Material online), which are from regions that so far have been understudied in previous investigations, such as India, Indonesia, Malaysia, and Pakistan (supplementary table S1, Supplementary Material online).

The publicly available data sets included WGS of 644 individuals (572 domestic sheep, 32 Asiatic mouflon, 6 bighorn sheep, 6 thinhorn sheep, 9 urial, 8 argali, 8 snow sheep, and 3 European mouflon), and they were derived from five sources: the NextGen Consortium, the International Sheep Genomics Consortium (ISGC), and three of our previous studies (Deng et al. 2020; Li et al. 2020; Chen et al. 2021). Summary information of these newly sequenced and publicly available samples, including population names, codes, geographic origins, sample sizes, and contributors, is detailed in supplementary table S1, Supplementary Material online. Coordinates of geographic origins of the populations were provided by the contributors or were assigned as the centroid of their known core distribution areas. Blood samples, ear or skin tissues were collected; ear and skin samples were preserved in 95% ethanol at −80°C.

### Resequencing and Variant Calling

DNA of the samples sequenced in this study was extracted using the DNeasy Blood & Tissue kit (QiaGen, Shanghai, China), including an RNase A treatment. DNA integrity was inspected on agarose gels and concentration was quantified using a Qubit 2.0 Fluorometer (Life Sciences, CA). At least 1µg of genomic DNA was used to construct a sequencing library according to the manufacturer’s specifications for the TruSeq Nano Sample Prep Kit (Illumina Inc., San Diego, CA). Briefly, the DNA was fragmented, end polished, A-tailed, and ligated with the full-length adapter. Fragments of 400–500 bp were selected, PCR amplified and purified using the AMPure XP system (Beckman Coulter, IN). The prepared libraries were assessed on an Agilent2100 Bioanalyzer and quantified using real-time PCR. Paired-end libraries that had average insert sizes of approximately 350 bp were sequenced on the Illumina HiSeq X Ten platform (Illumina.) by Berry Genomics Co. Ltd, (Beijing, China), yielding 150-bp reads with a target depth of ∼20-fold coverage per genome.

We obtained ∼39 Gb of raw sequences for per sample and used the FastQC v0.11.9 (https://www.bioinformatics.babraham.ac.uk/projects/fastqc/, last accessed April 22, 2019) for assessing a per-base sequence quality. Further processing and removal of low-quality bases and artifact sequences were implemented using the Trimommatic v.0.36 (Bolger et al. 2014). The high-quality 150-bp/100-bp paired-end reads were aligned to the sheep reference genome *Oar_rambouillet_v1.0*. (https://www.ncbi.nlm.nih.gov/assembly/GCF_002742125.1/, last accessed May 12, 2019), using the Burrows–Wheeler aligner (BWA mem) v.0.7.8 (Li and Durbin 2009) with default parameters. We then converted the mapping reads into bam files and sorted the files using the SAMtools. Duplicates were removed by the MarkDuplicates module in GATK v 4.1.2.0 (McKenna et al. 2010). We calculated whole-genome sequencing coverage and depth of each sample using the SAMtools v.1.9. We only kept samples with depth >10× to call short variations, and selected samples with depth >15× to identify SVs.

SNP and indels were called from the bam files by the GATK *HaplotypeCaller* module with the GATK best-practice recommendations (McKenna et al. 2010). Raw GVCFs with the samples called individually were merged using the CombineGVCFs and called for SNPs using the GenotypeGVCFs. We then selected the candidate SNPs and created the selected SNP data using the GATK module *SelectVariants*. To avoid potential false-positive calls, we implemented “VariantFiltering” of the GATK for the selected SNPs and INDELs using the best practice parameters “QUAL < 30.0 ‖ QD < 2.0 ‖ MQ < 40.0 ‖ FS > 60.0 ‖ SOR > 3.0 ‖ MQRankSum < −12.5 ‖ ReadPosRankSum < −8.0” and “QD < 2.0 ‖ QUAL < 30.0 ‖ FS > 200.0 ‖ ReadPosRankSum < −20.0,” respectively. We then filtered out nonbiallelic SNPs.

After the quality screening, all the identified SNPs were further annotated using SnpEff v4.3t (Cingolani et al. 2012) based on the gene annotations of the sheep reference genome *Oar_rambouillet_v1.0*. Locations for SNPs in various genic and intergenic regions as well as synonymous or nonsynonymous SNPs in exonic regions were annotated. Additionally, we enriched for SNPs specific for species, population, or geographic groups of populations using the SnpEff v4.3t. We annotated each of the significant species-, population- or region-specific enriched SNPs, in particular the nonsynonymous SNPs, using SnpEff v4.3t.

### SNP Validation

To check the confidence of SNPs called, we compared the SNPs identified with the *O. aries* dbSNP v.151 (http://www.ncbi.milm.nih.gov/SNP, last accessed July 10, 2021). In addition, we validated the SNPs in specific genes by the Sanger method of DNA sequencing. A total of 133 SNPs from 16 individuals of ten populations were genotyped by PCR and Sanger sequencing. The primers used for PCR were designed with FastPCR v6.7 (Kalendar et al. 2017; supplementary table S5, Supplementary Material online). The PCR reactions were carried out in 20 μl volume containing 10 μl of 2×taq PCR Master Mix (GeneBetter Biotech, Beijing, China), 0.8 μl (10 pmol/µl) for each forward and reverse primer (supplementary table S5, Supplementary Material online), 1μl DNA templates (30–100 ng/µl), and the remainder supplied with ddH_2_O. The reactions were performed by an Eppendorf 6331 Flexlid Mastercycler Nexus Cycler with conditions of an initial denaturation at 95°C for 15 min, followed by 35 cycles at 95°C for 30 s, annealing at 60°C for 90 s and extension at 72°C for 30 s, and then a final extension at 72°C for 7 min. The PCR products were sequenced by the Sanger method. All the reads were assessed manually and genotypes of each site were called by the sequencing peaks. Subsequently, we compared genotypes of each site identified by whole-genome resequencing and obtained by the Sanger sequencing for the same individuals.

### Structural Variant Calling

SVs were called from the sequences of 534 high-depth samples (>15×). We used three approaches of the Manta v1.3.2 (Chen et al. 2016), Delly v0.8.3 (Rausch et al. 2012), and smoove-0.2.6 (https://github.com/brentp/smoove, last accessed June 15, 2020) with default parameters to detect SVs such as deletions (DEL), inversion (INV), and duplications (DUP) with sizes of 50 bp–1Mb and translocations (TRA). We filtered all SVs based on the following criteria: 1) 50 bp ≤ SV≤1 Mb; and 2) the quality filtering parameters suggested by two approaches (flag PASS). The smoove-0.2.6 was implemented to combine the three SV data sets generated by the above three software and perform genotyping. The merged SV genotypes were retained following the criteria: 1) genotypes of SVs detected by all the three approaches were identical for at least two approaches; and 2) for SVs detected only by two out of the three approaches, their genotypes identified by smoove-0.2.6, or Manta v1.3.2 were kept.

### Genetic Diversity

We used the filtered set of 121.2 million high-quality SNPs for estimation of genetic diversity, population differentiation and LD decay metrics including expected heterozygosity (*H*_e_), nucleotide diversity (*π*), runs of homozygous (ROH), and LD decay. We summarized the numbers of homozygous and heterozygous SNPs against the reference genome. Values of *π* and *H*_e_ were estimated for nonoverlapping 1 Mb regions along the genome of each individual using the vcftools v0.1.17 (Danecek et al. 2011). We used PLINK v1.90p (Purcell et al. 2007) (–homozyg-density 50 –homozyg-window-het 1 –homozyg-window-kb 200 –homozyg-window-snp 50) to estimate the number (*N*_ROH_) and size (*S*_ROH_) of ROH and individual autozygosity (*F*_ROH_) (McQuillan et al. 2008).

Overall genetic differentiation across domestic populations measured by Weir and Cockerham’s estimator of *F_S_*_T_ (Weir and Cockerham 1984) was calculated using the vcftools v0.1.17 with 100 kb bins. *F*_ST_ values between populations or groups of populations were estimated with 220,350 unlinked SNPs using the gcta_1.93.2beta (Yang et al. 2011). The statistical significance of *F*_ST_ estimates was tested using the approach *t* test implemented in the *R* program (R Core Team 2020).

We examined the patterns of LD decay within each species or population by random selection of three individuals in order to avoid biases by difference in sample sizes and using the vcftools v0.1.17 to extract individual data for LD analysis. Pairwise LD estimates were measured as parameter *r*^2^ with a maximum distance of 300 kb using the PopLDdecay v3.41 (Zhang et al. 2019).

### Population Genetics Analysis

To remove the potential impact of close relatives on estimation, we calculated the pair-wise KING-robust kinship estimator using the KING v2.2.5 (Manichaikul et al. 2010). We excluded one of each sample pair showing close relatedness with kinship coefficient > 0.0884 (e.g., duplicates/monozygotic twins, 1^st^-degree and 2^nd^-degree relatives) (Manichaikul et al. 2010), which resulted in 714 unrelated individuals for genetic diversity analyses (supplementary table S13, Supplementary Material online). We further filtered the SNP data set with a MAF < 0.05, Hardy–Weinberg equilibrium <0.001 and a proportion of missing genotypes >10%. We implemented LD pruning with the PLINK option “–indep-pairwise 50 5 0.2.” After the filtering and LD pruning, 24,363,608 SNPs were retained for population genetics analyses.

First, we calculated the identity-by-state (IBS) genetic distance matrix between the individuals using the PLINK v1.90 (–distance 1-ibs) and visualized by an unrooted neighbor-joining (NJ) phylogenetic tree using the SplitsTree5 v5.1.7-beta (Huson and Bryant 2006). PCA was performed using the smartpca function built in the EIGENSOFT v.6.0.1 (Patterson et al. 2006) with default settings. Furthermore, model-based clustering was performed using the sNMF v1.2 with the number of cluster *K* from 1 to 20. The optimal *K* was determined by the minimal value of the cross-entropy criterion of sNMF.

### Demographic Inference

We used the PSMC model (Li and Durbin 2011) to reconstruct changes in historical effective population size (*N*_e_) through time. Analyses within species or populations were based on the three individuals with the highest mean sequencing coverage per population. The psmc function built in the PSMC package was used to estimate *N*_e_ with the parameters “–N25 –t15 –r5 –p *‘*4 + 25*2 + 4 + 6.” We used a mutation rate of 1.51 × 10^-8^ mutations per nucleotide site per generation (Chen et al. 2019) and a generation time of 3 years (Zhao et al. 2017; Alberto et al. 2018) to convert the resulting scaled values into years and individuals.

We also inferred the dynamics of recent historical *N*_e_ during the past 1,000 to 100,000 years using the SMC++ v1.14.0.dev0 (Terhorst et al. 2017). We selected 7–10 high-depth unrelated individuals in each of the six geographically differentiated genetic groups (African, European, Central-and-East Asian, the Middle Eastern, South-and-Southeast Asian, and American populations). Bi-allelic SNPs of the corresponding individual were extracted from the raw *vcf* files. We further masked low-complexity regions of the reference genome *Oar_rambouillet_v1.0* following the method of Li Heng (http://lh3lh3.users.sourceforge.net/snpable.shtml, last accessed October 20, 2020). The mutation rate was set at 1.51 ×10^-8^ mutations per nucleotide site per generation (Chen et al. 2019). All other parameters were set to their default values while the same mutation rate (Chen et al. 2019) and generation time (Zhao et al. 2017; Alberto et al. 2018) were applied to the final representation of recent demographic events of *N*_e_.

Historical effective population size (*N*_e_) was also estimated using the program SNeP v.1.11 (Barbato et al. 2015) with default settings. The program SNeP estimates *N*_e_ based on the relationship between *r*^2^, *N*_e_, and *c* (recombination rate) following the equation *E* (*r*^2^) = 1/(1 + 4 *N*_e_*c*) (Sved 1971). Only the SNPs with nonmissing genotypes and MAF > 0.05 were included in this estimation. We used the different SNP marker distance bins in the LD (*r*^2^) analysis to estimate *N*_e_ at *t* = 1/2*c* generation ago.

### Migration and Divergence Modeling

#### Data Set

Demographic history of sheep postdomestication was reconstructed using the fastsimcoal v2.6 (Excoffier et al. 2013). Forty-eight high-depth (15.1–22. 6×) domestic samples from Africa (*n* = 10), Europe (*n* = 10), Central-and-East Asia (*n* = 10), South-and-Southeast Asia (*n* = 10), and the Middle East (*n* = 8) were selected as the representative samples of different geographic and genetic origins based on their population history and results of the population genetics analyses (supplementary fig. S29 and table S50, Supplementary Material online).

For these statistical modeling analyses, we first selected 6,477,008 high-quality autosomal SNPs that: 1) are located within intergenic regions and 2) are outside CpG islands, defined according to the UCSC annotation (https://hgdownload.soe.ucsc.edu/hubs/GCF/002/742/125/GCF_002742125.1/bbi/, last accessed October 27, 2020), 3) do not have missing genotypes, 4) have a depth >10× in all samples, and 5) have an ancestral state inferred from the goat and cattle reference genomes. We then generated the multidimensional SFS file using the python script vcf2sfs.py (https://github.com/marqueda/SFS-scripts, last accessed November 12, 2020). In order to calculate confidence intervals for the parameters, we resampled 2,647 ∼ 1-Mb blocks on the autosomes with similar cumulative genomic length as in the observed data (∼2,234 Mb) and generated 100 multidimensional SFS data sets.

#### Statistical Modeling

We investigated the migration and divergence of the South-and-Southeast Asian populations and estimated the time of their main expansions and gene flows among populations in different continents. On the basis of the model inferred by Lv et al. (2015) and Zhao et al. (2017), three alternative models without gene flow among populations from the different main geographic regions were tested for their origins and demographic history of South-and-Southeast Asian populations (supplementary fig. S13, Supplementary Material online): scenario I: descent of the ancestral Middle Eastern breeds; scenario II: descent of Central-and-East Asian breeds; and scenario III: originated from early admixture between Central-and-East Asian and the Middle Eastern breeds. We assumed a ghost population as the common ancestor of modern domestic sheep that was present in the Middle East after domestication, but before their expansions to other regions.

In the optimal model, asymmetric gene flow between modern populations from neighboring geographical regions were added, such as between African and European breeds, between African and the Middle Eastern breeds, between the Middle Eastern and South-and-Southeast Asian breeds, between the Middle Eastern and Central-and-East Asian populations, between South-and-Southeast Asian and Central-and-East Asian populations, but not between the ghost and modern populations (supplementary fig. S30, Supplementary Material online).

#### Maximum-Likelihood Parameter Estimation

We estimated the maximum likelihood values for scenarios I, II, and III, independently, after performing 1,000,000 simulations, 65 conditional maximization cycles, and 100 optimization runs starting from random initial conditions. We used approaches described in Choin et al. (2021) and Malaspinas et al. (2016) to optimize the fit between the expected and observed SFS (supplementary fig. S31, Supplementary Material online). We calculated the maximum likelihood of each model with all entries of SFS for the first 25 cycles (-l 25) and then optimized the maximum likelihood of each model for the remaining 40 cycles (-L 65).

#### Nonparametric Bootstrapping Analysis

For the optimal model, we estimated confidence intervals for the parameters with nonparametric bootstrapping approach using the 100 multidimensional *SFS* data sets. We re-estimated parameters using the fastsimcoal v2.6 with same settings as for the original observed data set. We performed 20 replicate runs per bootstrap data set starting from random initial conditions. The 95% CIs for each parameter were obtained using the parameter estimates of all bootstrapping replicates with the *R* boot package. All analyses were implemented using the fastsimcoal v2.6 with the same mutation rate (Chen et al. 2019) and generation time (Zhao et al. 2017; Alberto et al. 2018).

### Interspecies Introgression Analysis

#### *D* Statistics

We tested for the presence of interspecific genetic introgression from wild relatives into domestic populations using different statistical analyses such as *D* statistics (Patterson et al. 2012) and *f*_d_ statistics (Martin et al. 2015). We calculated the *D* statistics for the four-taxon model (W, X, Y, Z) using the function *qpDstat* implemented in the AdmixTools v7.0.1 (Patterson et al. 2012). We used Menz sheep, a domestic population which did not show any signal of wild introgression in the admixture analysis, as the reference population (W), individual populations of domestic sheep as the target population (X), and each wild species as the donor (Y). We assumed that outgroup (*Z*) was fixed for the ancestral alleles when the target population was snow sheep, bighorn, or thinhorn sheep, whereas bighorn sheep was used as the outgroup (Z) when Asiatic mouflon, European mouflon, urial, or argali was treated as the donor population. A value of |*Z*| > 3 was considered to support an influence of the donor wild species and tested domestic populations.

#### Localization and Quantification of Introgression

In order to quantify the introgressed proportions of wild relatives in the genomes of domestic sheep and map the introgressed loci at the chromosome-wide level, we further conducted *f*_d_ analysis (Martin et al. 2015) based on the results of *D* statistics. Statistics *f*_d_, a modified version of *f* estimator (Green et al. 2010), is prone to extreme values in the genomic regions of low diversity, providing an accurate and robust method for detecting and quantifying the introgressed loci (Martin et al. 2015). The domestic populations with |*Z* scores| > 3 were selected as potential recipient populations. For each individual in the recipient populations, we used *f*_d_ to quantify the proportion of introgression as well as to identify the introgressed regions. For the windows with *f*_d_ < 0, the *f*_d_ statistic doesn’t have biological meaning. Therefore, the *f*_d_ values were converted to 0. We then calculated *P* value of each *f*_d_ by *Z* transform, and further corrected the *P* values with the Benjamini-Hochberg’s FDR method (Benjamini and Hochberg 1995).

Considering the distribution of introgressed tract lengths and to minimize nonindependences among adjacent windows and ensure sufficient resolutions, we used ABBABABAwindows.py to calculate the *f*_d_ statistic using sliding windows of 100 kb with a step of 50 kb across the genomes (Martin et al. 2016). We set the minimum SNPs in each window of 500 with the option “-w 100000 -m 500 -s 20000.”

We used the popgenWindows.py to calculate *π*, *F*_ST_, and *d*_xy_ as a measure of genetic variation, genetic differentiation, and absolute divergence, with the same sliding windows parameters used in the *f*_d_ analysis, for the following two groups of comparisons: donor versus recipient population (-p donor -p recipient) and donor versus reference population (-p donor -p reference) (Martin et al. 2015). We converted the meaningless or noisy *f*_d_ value (*D* < 0, or *D* > 0, but *f*_d_ > 1) to zero because theoretically only a positive *f*_d_ statistic value was considered as evidence of introgression from population *P*3 into *P*2 under a four-taxon topology ((*P*1, *P*2), *P*3, O) (Martin et al. 2015). We estimated the *f*_d_ statistic value using Menz sheep as *P*1, individual domestic populations as *P*2, individual wild species as *P*3, and ancestral alleles (when *P*3 was snow sheep, bighorn sheep, or thinhorn sheep) or bighorn sheep (when *P*3 was Asiatic mouflon, European mouflon, urial or argali) as O in the analysis.

#### Incomplete Lineage Sorting

We inferred that a genomic tract in the gene *HBB* was introgressed from one of the wild sheep species into Changthangi sheep. To verify that the target genomic tract is the result of introgression, we calculated the probability of the alternative scenario of ILS. The expected length of a shared ancestral sequence is *L* = 1/(*r*×*t*), where *r* is the recombination rate per generation per base pair and *t* is the branch length between argali/Asiatic mouflon/European mouflon/urial and domestic sheep since their divergence. The probability of a length of > *m* is 1 − GammaCDF (*m*, shape = 2, *r* = 1/*L*), where GammaCDF is the Gamma distribution and *m* is the length of introgressed tracts (Huerta-Sanchez et al. 2014).

### Detection of Selective Signatures

#### Selective Signatures in Domestic Sheep during Improvement

To identify the genomic signatures of selection in domestic sheep, we calculated the average SNP *F*_ST_ values (Weir and Cockerham 1984) using the VCFtools v1.17. All genomic regions in 50-kb sliding windows were scanned across genomes of the 158 populations using 10-kb steps. Regions with top 1% of the average *F*_ST_ distribution were identified as selection signatures. Furthermore, we examined the changes in genetic diversity (as measured in *π*) due to artificial selection. The ROD values in 10-kb nonoverlapping windows were calculated between landraces and improved populations using the formula: ROD = 1 − *π*_improved populations_/*π*_landraces_ (Wu et al. 2020).

#### Selection Associated with Fleece Fiber Diameter

We tested for the potential selective sweeps in the pairwise comparisons between groups of hair, coarse-wool, medium-wool, and fine-wool populations (supplementary table S32, Supplementary Material online). A XP-CLR test was implemented to scan the genomes for selective sweeps using the program XP-CLR v1.0 (Chen et al. 2010). The SNPs with less than 10% missing data were allowed (“–max-missing 0.9”) in the analysis. We calculated XP-CLR scores using the grid points spaced by 2 kb, a maximum of 200 SNPs in a window of 0.5 cM, and the down-weighting contributions of highly correlated SNPs (*r*^2^ > 0.95) with the parameters (-w1 0.005 200 2000 2 -p0 0.95). We considered genomic regions with the top 1% region-wise XP-CLR scores as candidate selective sweeps. Moreover, we conducted a selection test based on the SVs by calculating the statistic *F*_ST_ (Bertolotti et al. 2020). For each SV, we calculated the *F*_ST_ values between the above-mentioned pairwise comparisons of groups of populations with different fleece fiber diameters using the VCFtools v0.1.17. To identify the significance of each *F*_ST_ value of individual SVs, we generated 200 randomly sampled *F*_ST_ values, and obtained *P* values of per SV by calculating the proportion of *F*_ST_ values obtained in the 200 random distributions higher than the *F*_ST_ estimates in the observed distribution (Bertolotti et al. 2020). We selected the SVs with the *P* < 0.01 as the candidate selective SVs.

#### Selection for Particular Phenotypes

To identify alleles that are either close to fixation or already reached fixation in particular populations due to past selection on phenotypes such as dwarf (e.g., Ouessant sheep), we calculated CLR scores using the SweeD (Pavlidis et al. 2013). We used 10-kb nonoverlapping sliding windows in the tested Ouessant population. Empirical *P* values were calculated for CLR windows and the top 5% of windows were considered as candidate signatures of selection.

#### Selection Related to High-Altitude Adaptation

We conducted PBS analysis (Yi et al. 2010) to identify the selection signatures associated with high altitude adaptation. Because we detected the introgression signals in *HBB* and *HBBC* genes from argali to Changthangi sheep in Ladakh (*ca.* 4,000 m above sea level), we used Changthangi sheep (CHA) as the representative high-altitude population, East Friesian sheep (EFR, living around the sea level) as the low-altitude population, and Hu sheep (HUS, living around the sea level) as the reference low-altitude population to perform the PBS analysis. We used *R* scripts from the github (https://github.com/genevol-usp/EvoGen_course, last accessed January 5, 2021) to estimate pairwise population *F*_ST_ values between three comparisons of Changthangi versus East Friesian sheep, Changthangi versus Hu sheep and East Friesian versus Hu sheep. PBS value was calculated following the equation: PBS = ((−log(1 − *F*_ST_.EFR.CHA)) + (−log(1 − *F*_ST_.CHA.HUS)) − (−log(1 − *F*_ST_.EFR.HUS)))/2 (Yi et al. 2010). The PBS values with 99^th^ empirical thresholds were identified as outlier signals under selection.

### Gene Annotation, GO, and KEGG Analyses

We annotated genes associated with the candidate SNPs, CNVs, or genomic regions using the sheep reference assembly *Oar_rambouillet_v1.0*. GO term enrichment and KEGG pathway analyses of a candidate gene set were carried out by a statistical overrepresentation test with the default setting in the Database for Annotation, Visualization, and Integrated Discovery (DAVID) database v6.8 (Huang et al. 2009). Categories with at least six genes and the threshold of adjusted *P* value < 0.05 after the Bonferroni correction were considered as significantly enriched terms and pathways.

### Histological Analysis

To examine the numbers and the ratio of secondary to primary wool follicles in the important development stage of embryonic skin (embryo 85–90 days), fixed dorsal skin samples for coarse-wool (e.g., Wadi sheep) and fine-wool (e.g., Chinese Merino) sheep were dehydrated with gradient alcohol, processed in paraffin and cut into histological longitudinal sections of 5μm. The dorsal skin sections were processed by dewaxing, stained with H&E, and photographed for the identification of the secondary and primary wool follicles.

### RNA-seq and miRNA-seq Analysis

#### RNA-seq Analysis

RNA-seq data for samples from eight fine-wool and medium-wool populations (e.g., Aohan fine wool, Chinese Merino, Churra, Erdos fine -wool, Gansu Alpine fine wool, Rambouillet, Super fine wool Merino, and Texel sheep) and seven coarse-wool populations (e.g., Bashibai, Hu, Kazakh, Min Xian Black Fur, Small-tailed Han, Tan, and Tibetan sheep) were retrieved from public database (supplementary table S45, Supplementary Material online). RNA-seq reads were quality filtered with the FastQC v0.11.9 (https://www.bioinformatics.babraham.ac.uk/projects/fastqc/, last accessed April 22, 2019). Low-quality ends of the reads were removed using Cutadapt (Martin 2011). Reads from each sample were aligned to the *Oar rambouillet* v1.0 reference genome (https://www.ncbi.nlm.nih.gov/assembly/GCF_002742125.1, last accessed May 12, 2019) using the TopHat (Trapnell et al. 2012) with default options. Transcript quantification, normalization, and assembly were implemented using the Cufflinks (Trapnell et al. 2012). The FPKM of *IRF2BP2* gene (chr25:7,067,974–7,071,785) was then calculated to quantify its expression level.

#### Prediction of the Target miRNA for IRF2BP2

Three different programs Targetscan (Agarwal et al. 2015), miRBase (Kozomara et al. 2019), and miRDeep2 (Mackowiak 2011) were used to jointly predict the target miRNA based on the mRNA sequence of the *IRF2BP2* gene (fine-wool sheep: 5′-_**…**_GUUGGUUAC**G**UAAUACACA_**…**_-3′ and coarse-wool sheep: 5′-_**…**_GUUGGUUAC**A**UAAUACACA_**…**_-3′). The program Targetscan was used to analyze the evolutionary conservation of the miRNAs bound to the 3′-UTR of *IRF2BP2*, and the binding sites of *IRF2BP2* and corresponding miRNAs in different species (e.g., sheep, cattle, and goat). We used the miRBase database to obtain miRNA sequences of different species, evaluated site conservation, and selected miRNAs whose binding sites were relatively conserved in sheep and other species. The target identified by all the three programs above was considered to be the potential target miRNA for *IRF2BP2*.

#### miRNA-seq Analysis

Small RNA raw data were obtained from public database and filtered through the Cutadapt (Martin 2011) by excluding adaptor sequences, low-quality reads, and sequences <18 nt or >30 nt (supplementary table S47, Supplementary Material online). Reads were mapped to the *Oar rambouillet v1.0* reference genome and aligned with mature miRNA sequences in the miRBase database to identify known miRNAs. The expression levels of miRNAs in coarse-wool and fine-wool sheep breeds were estimated by using the sRNABench (Barturen et al. 2014), which normalized reads count number of each miRNA reads per million (RPM) using the following formula: RPM = (miRNA reads number/total mapped reads per library) × 1,000,000.

#### Cell Lines and Dual Luciferase Reporter Assay

HEK293T cells were used to validate the *IRF2BP2* target. Cells were cultured in the Dulbecco′s modified eagle medium plus 10% fetal bovine serum and penicillin/streptomycin. The cells were propagated at 37°C, 90% of air humidity and with 5% of CO_2_. All reagents used for cell culture were provided by the Invitrogen/Gibco (Beijing, China). The cells were seeded into 24-cell plates and transfected 24 h later with Lipofectamine 2000. WT/mutated *IRF2BP2* 3′-UTR and miRNAs mimics were generated according to the mRNA and miRNA sequences (supplementary table S46, Supplementary Material online).

HEK293T cells were transfected with Lipofectamin 2000 in 24-well plates. The psiCHECK-2 vector containing WT or mutated (MU) 3′-UTR of *IRF2BP2* vector was cotransfected into HEK293T with mimic or negative control of miRNA (i.e., WT + miRNA mimic, WT + negative control, MU + miRNA mimic, and MU + negative control). One day after the transfection, the cells were lysed, and then the Renilla and Firefly substrates were added using the dual-luciferase reporter kit (Promega, WI) following the manufacturer’s recommendations. The Renlilla luciferase signals were normalized to firefly luminescence. These experiments were repeated three times. A two-sided Student’s *t*-test was performed to evaluate statistical significance of the differences between the miRNA negative control and miRNA mimics.

## Supplementary Material

Supplementary data are available at *Molecular Biology and Evolution* online.

## Supplementary Material

msab353_Supplementary_Data
