# Supplementary material for: Whole-Genome Resequencing of Worldwide Wild and Domestic Sheep Elucidates Genetic Diversity, Introgression, and Agronomically Important Loci
Source: Mol Biol Evol. 2021 Dec 10;39(2):msab353. doi: 10.1093/molbev/msab353 (PMC8826587; doi:10.1093/molbev/msab353)
Supplement: msab353_Supplementary_Data [file msab353_supplementary_data.zip › Supplementary_Information (6).docx]

**Supplementary Material for**

**Whole-genome resequencing of worldwide wild and domestic sheep elucidates genetic diversity, introgression and agronomically important loci**

Feng-Hua Lv^1,41^, Yin-Hong Cao^2,3,41^, Guang-Jian Liu^4,41^, Ling-Yun Luo^1^, Ran Lu^1^, Ming-Jun Liu^5^, Wen-Rong Li^5^, Ping Zhou^6,7^, Xin-Hua Wang^6,7^, Min Shen^6,7^, Lei Gao^6,7^, Jing-Quan Yang^6,7^, Hua Yang^6,7^, Yong-Lin Yang^6,7^, Chang-Bin Liu^6,7^, Peng-Cheng Wan^6,7^, Yun-Sheng Zhang^6,7^, Wen-Hui Pi^6,7^, Yan-Ling Ren^8^, Zhi-Qiang Shen^8^, Feng Wang^9^, Yu-Tao Wang^10^, Jin-Quan Li^11^, Hosein Salehian-Dehkordi^2,3^, Eer Hehua^12^, Yong-Gang Liu^13^, Jian-Fei Chen^1^, Jian-Kui Wang^1^, Xue-Mei Deng^1^, Ali Esmailizadeh^14^, Mostafa Dehghani-Qanatqestani^14^, Hadi Charati^14^, Maryam Nosrati^15^, Ondřej Štěpánek^16^, Hossam E. Rushdi^17^, Ingrid Olsaker^18^, Ino Curik^19^, Neena A. Gorkhali^20^, Samuel R. Paiva^21^, Alexandre R. Caetano^21^, Elena Ciani^22^, Marcel Amills^23,24^, Christina Weimann^25^, Georg Erhardt^25^, Agraw Amane^26,27^, Joram M Mwacharo^28^, Jian-Lin Han^29,30^, Olivier Hanotte^27,31,32^, Kathiravan Periasamy^33^, Anna M. Johansson^34^, Jón H. Hallsson^35^, Juha Kantanen^36^, David W. Coltman^37^, Michael W. Bruford^38,39^, Johannes A. Lenstra^40^, Meng-Hua Li^1*^

*Corresponding author: [menghua.li@cau.edu.cn](mailto:menghua.li@cau.edu.cn)

**This PDF file includes:**

Supplementary Figs. S1 to S31

Supplementary Tables S1 to S50 (see the excel files)

**Supplementary Figures**

**
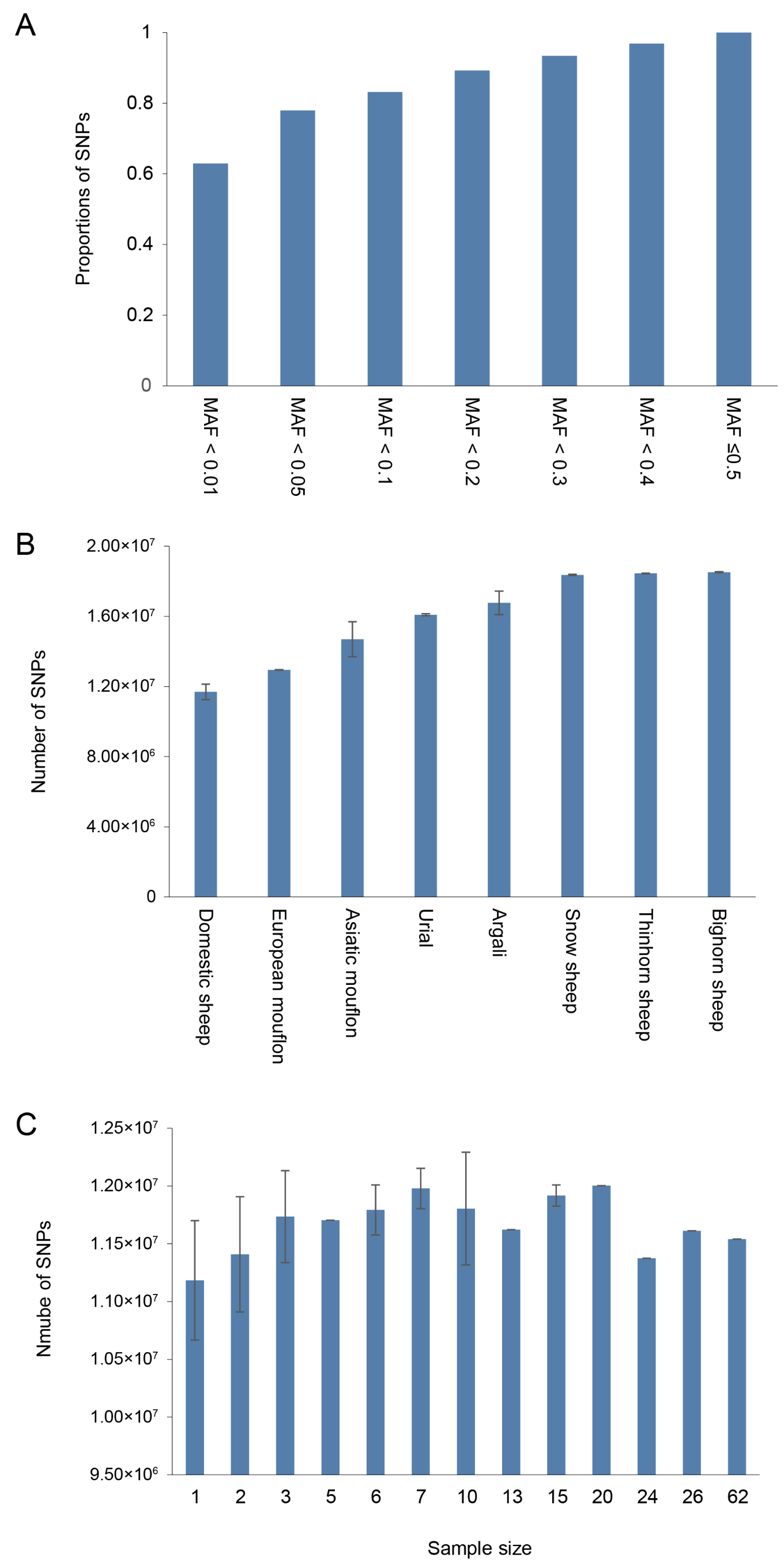
**

**Supplementary Fig. S1. Number of SNPs in 810 wild and domestic sheep samples**. **A)** At different levels of minor allele frequencies (MAF); **(B)** In each species; **(C)** In the populations with different sample sizes.


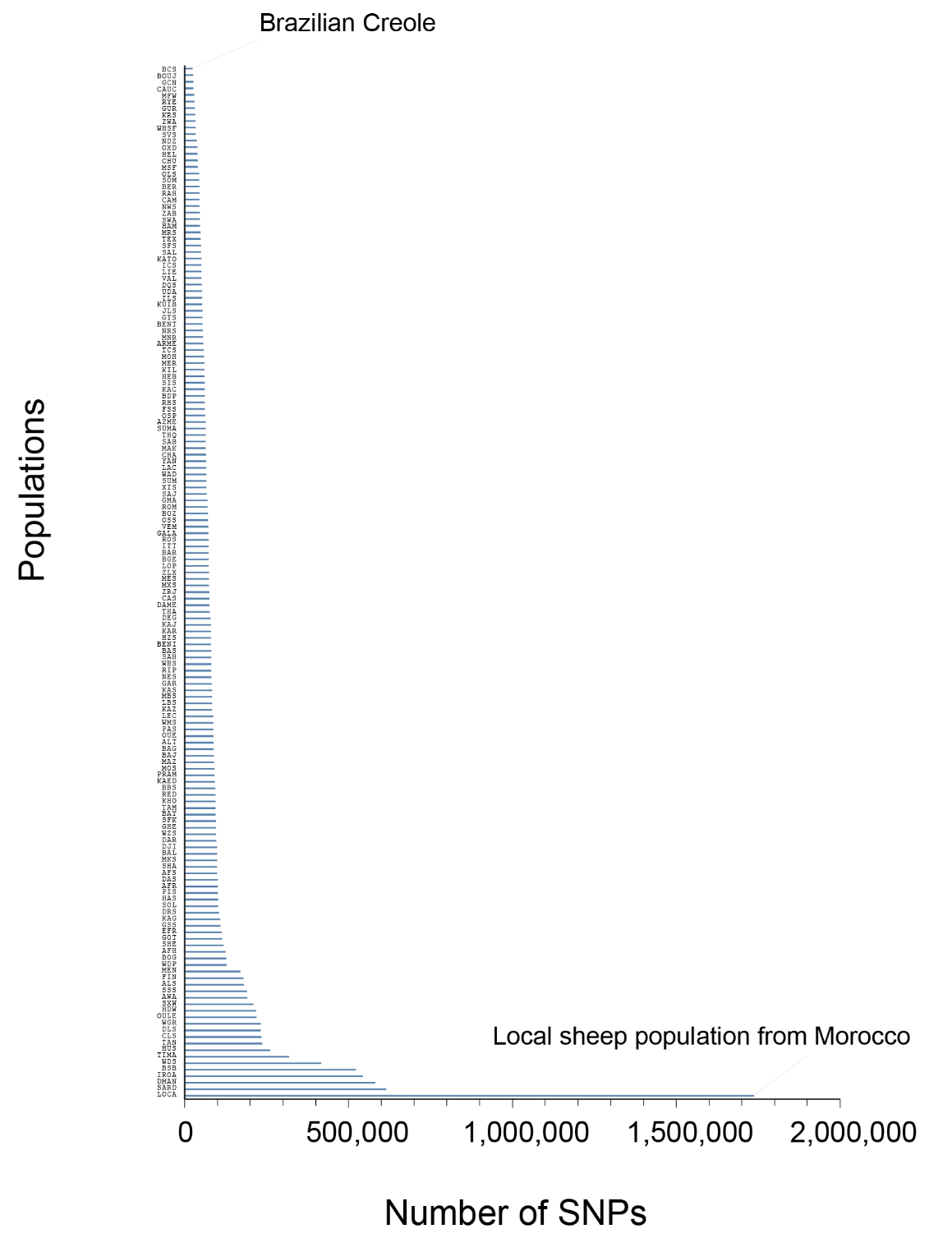


**Supplementary Fig. S2. Number of SNPs in each population of domestic sheep**.


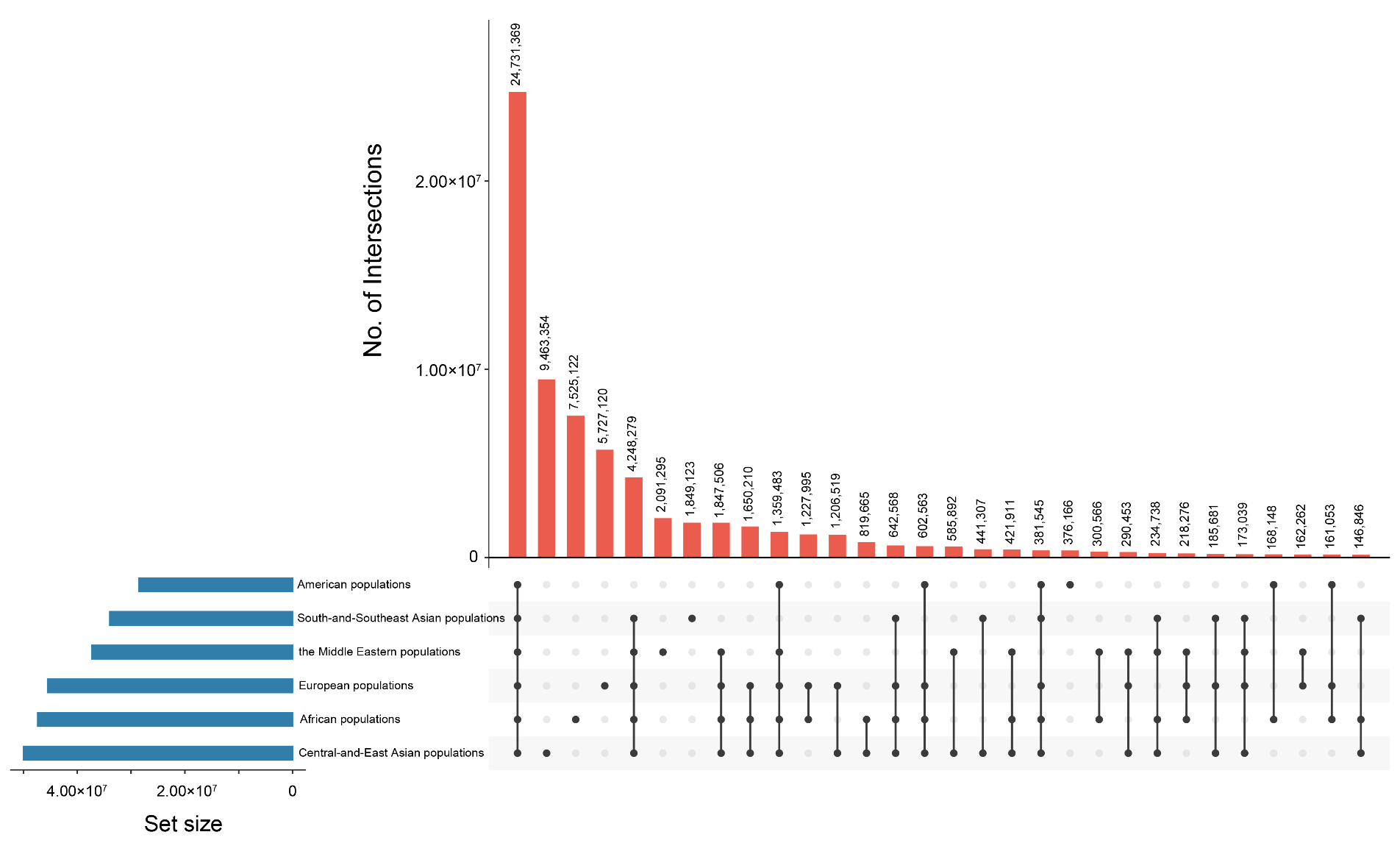


**Supplementary Fig. S3. UpSet plot of intersection of the number of SNPs in domestic sheep populations from six major geographic regions**.


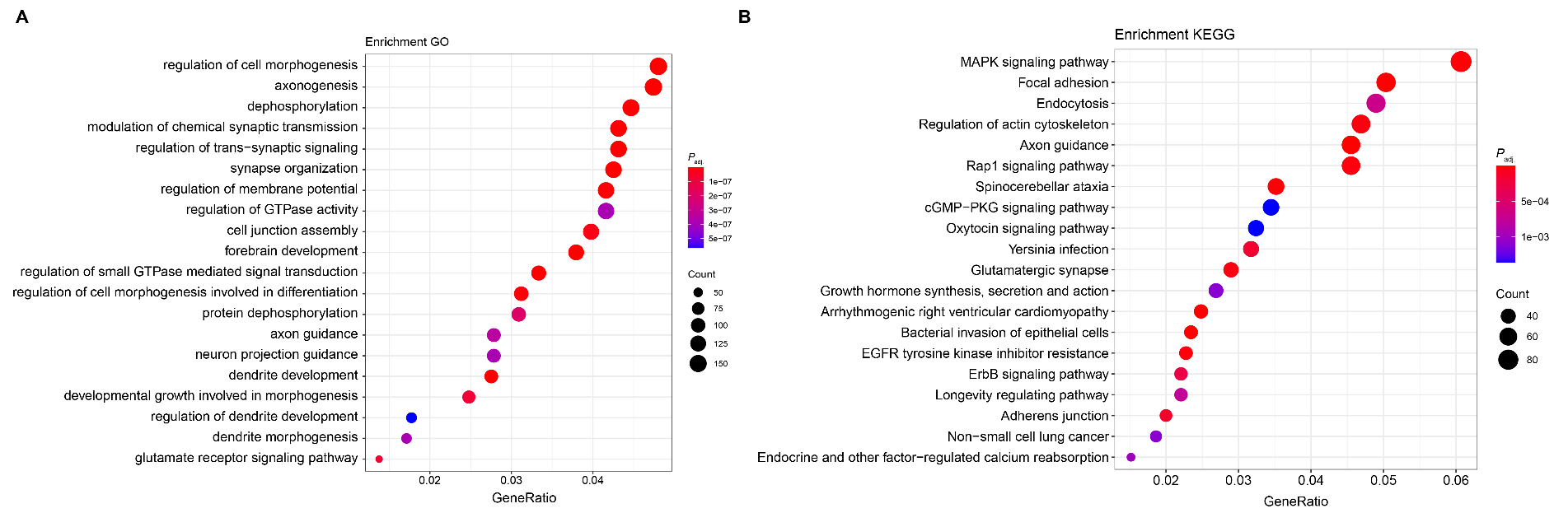


**Supplementary Fig. S4. Enrichment analyses of functional genes overlapped with structure variations (SVs) unique in domestic sheep**. **(A)** GO analysis (*P*_adj._< 0.01); **(B)** KEGG pathways analysis (*P*_adj._ < 0.01).


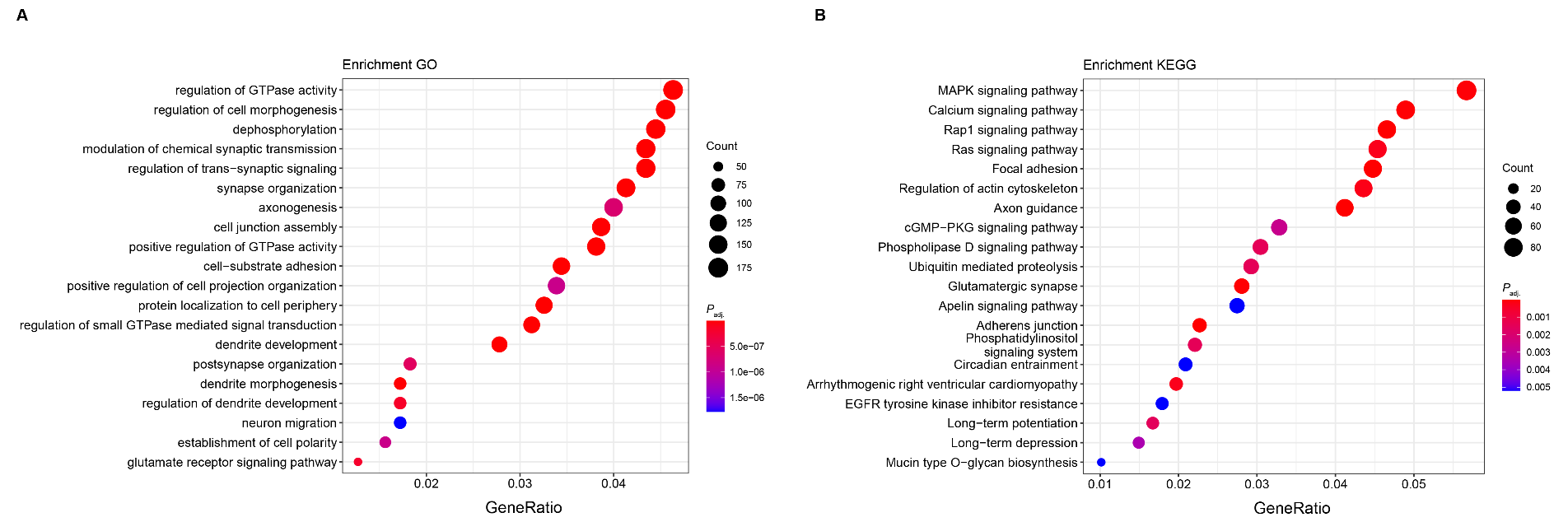


**Supplementary Fig. S5.** **Enrichment analyses of functional genes overlapped with structure variations (SVs) unique in wild sheep species**. **(A)** GO analysis (*P*_adj._< 0.01); **(B)** KEGG pathway analysis (*P*_adj._ < 0.01).


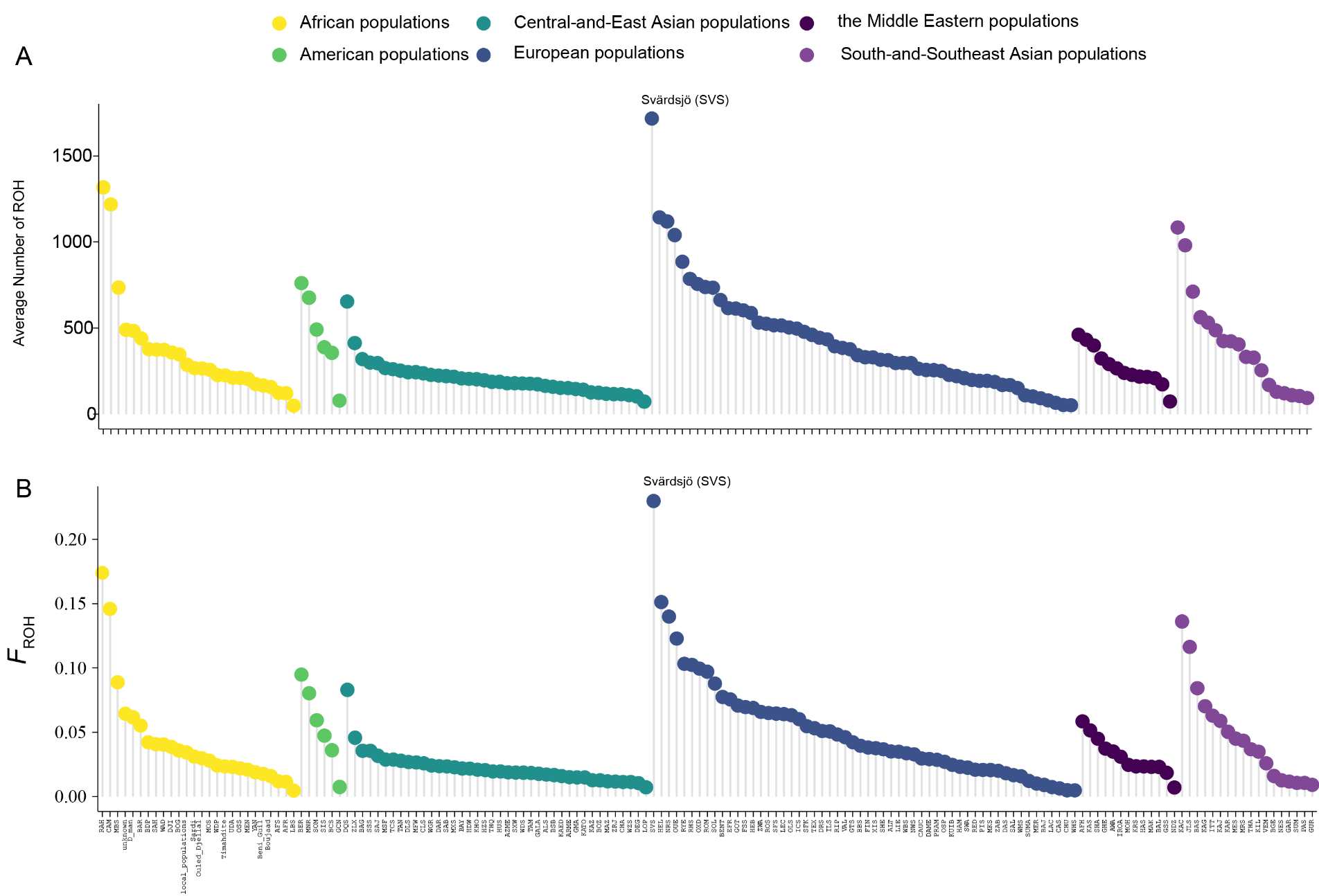


**Supplementary Fig. S6. Summary statistics of ROH (runs of homozygosity)**. **(A)** Average number of ROH in each population of domestic sheep; **(B)** The genomic inbreeding coefficients (*F*_ROH_) in each population of domestic sheep.


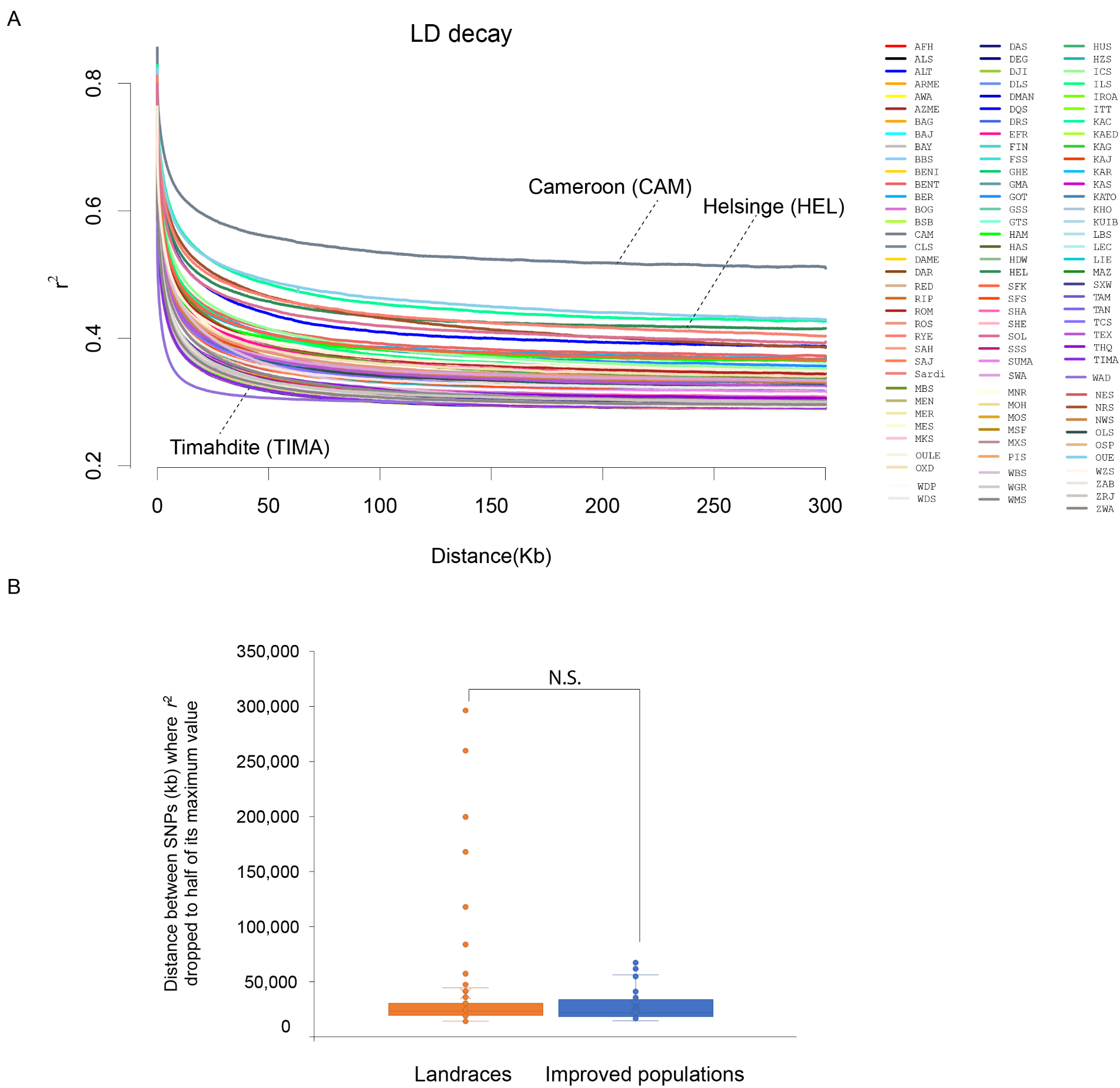


**Supplementary Fig. S7. Levels of linkage disequilibrium (LD) in each population of domestic sheep**. **(A)** Decay of LD with the distances between pairwise SNPs; **(B)** Level of LD half the decay distance, the point at which the observed *r*^2^ between sites decays to less than half the maximum *r*^2^ value.


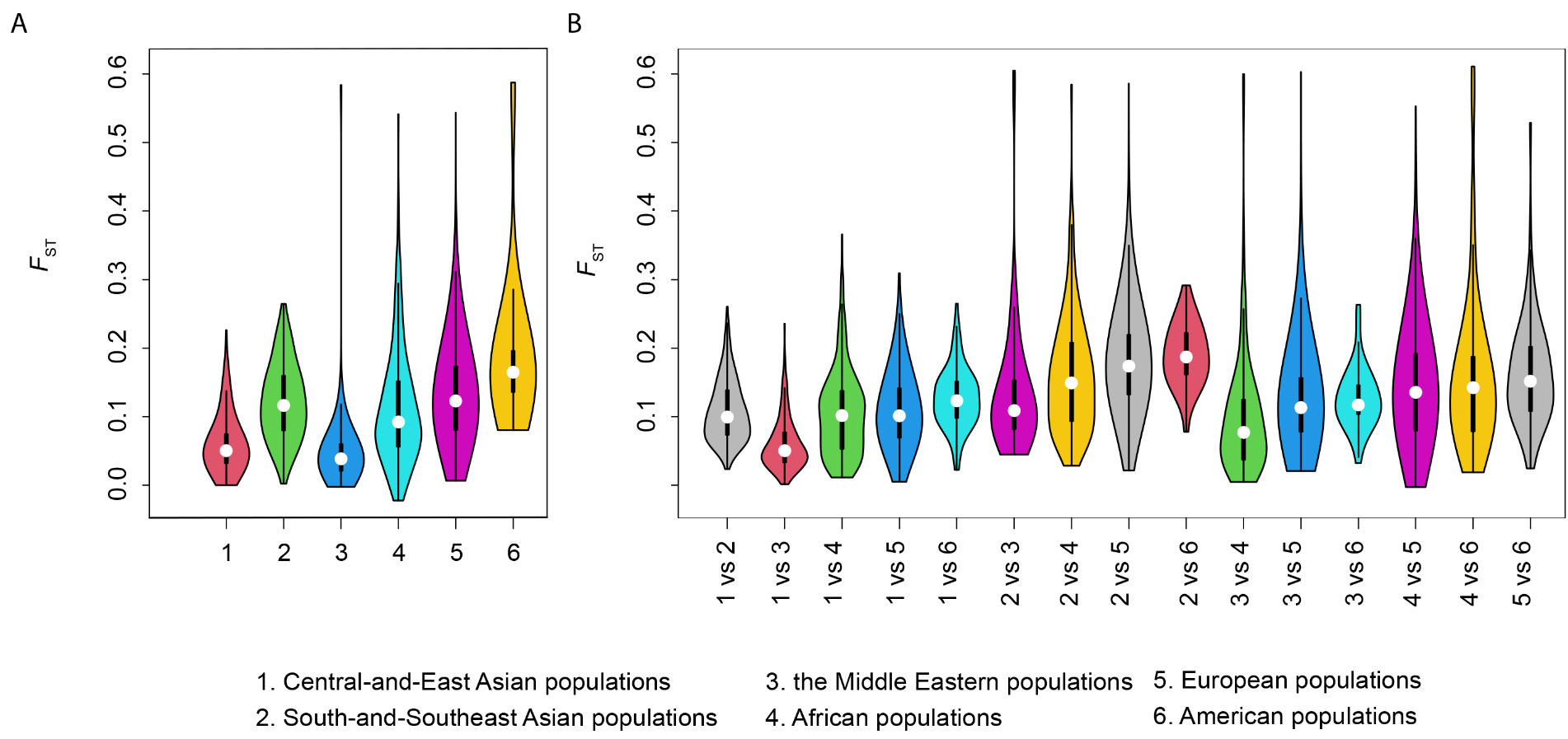


**Supplementary Fig. S8. Average estimates of genetic differentiation (*F*_ST_) between pairwise populations**. **(A)** Within the same geographic groups; **(B)** From different geographic groups.


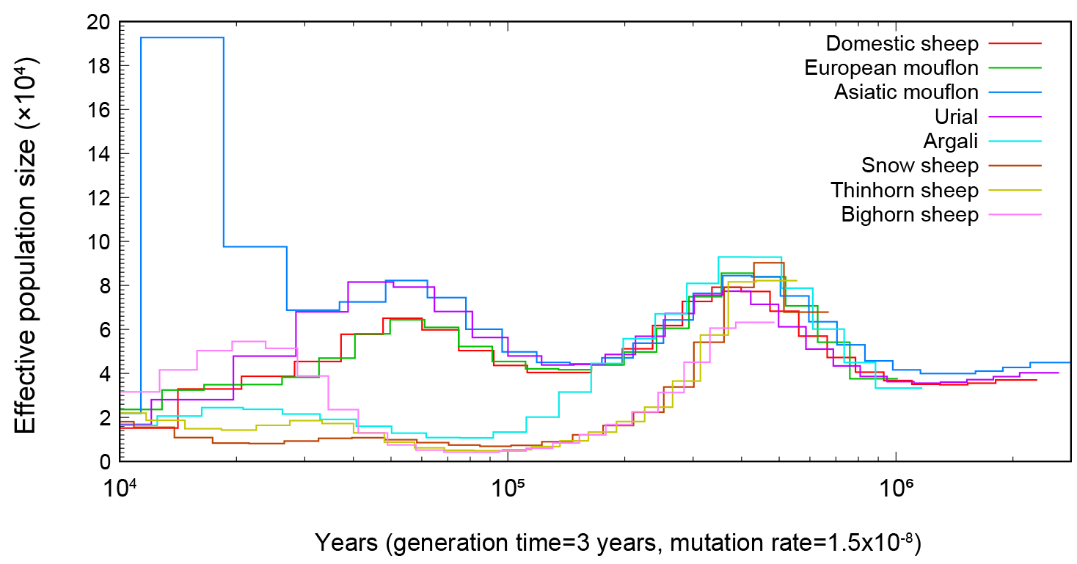


**Supplementary Fig. S9. Pairwise Sequentially Markovian Coalescent (PSMC) analysis.** Inferred historical population sizes of eight wild and domestic species by pairwise sequential Markovian coalescent analysis.


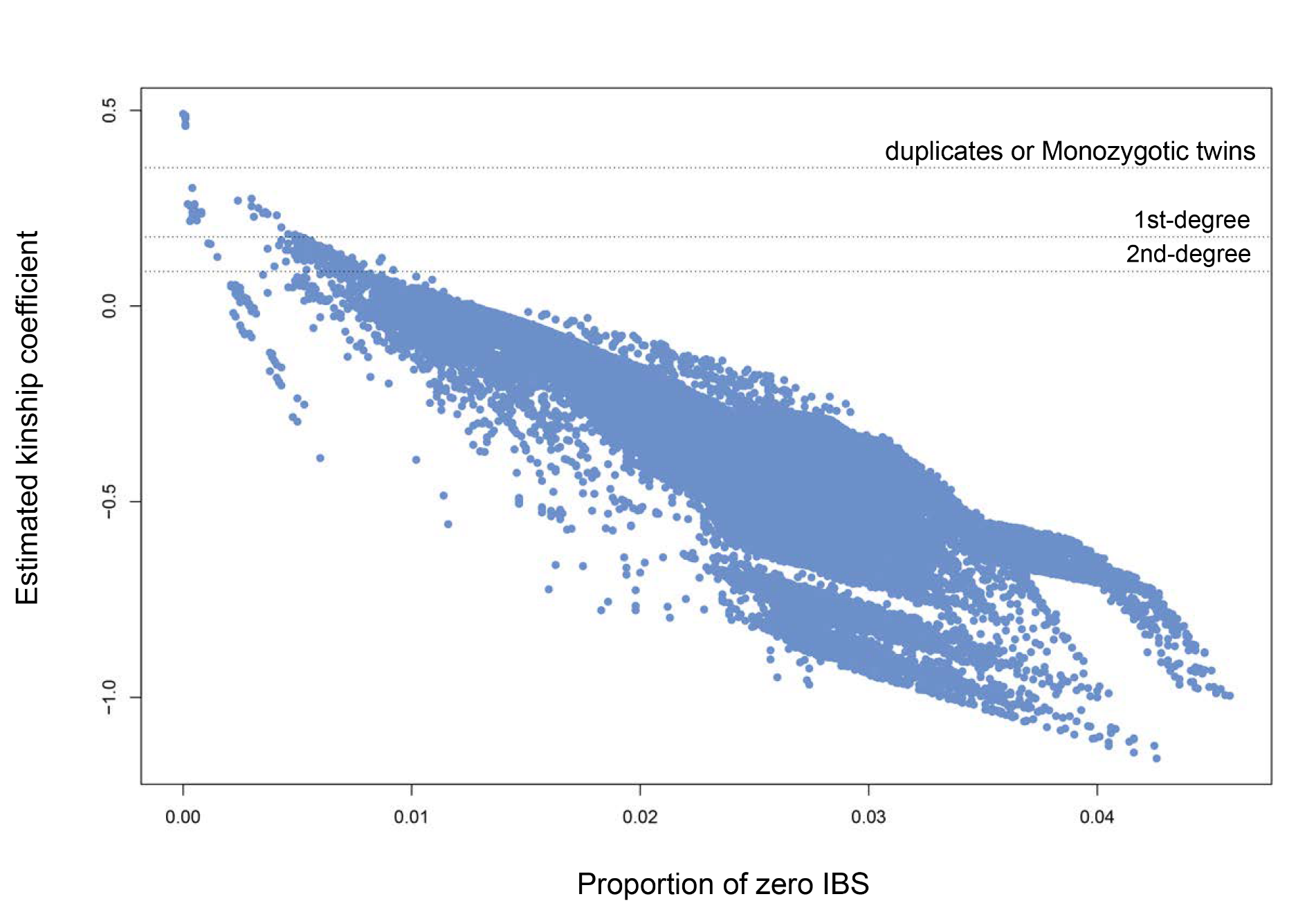


**Supplementary Fig. S10. Pairwise relatedness among individuals of domestic sheep**. Kinship coefficients and the proportions of markers that share no alleles (IBS0) were estimated by the KING (Manichaikul et al. 2010).


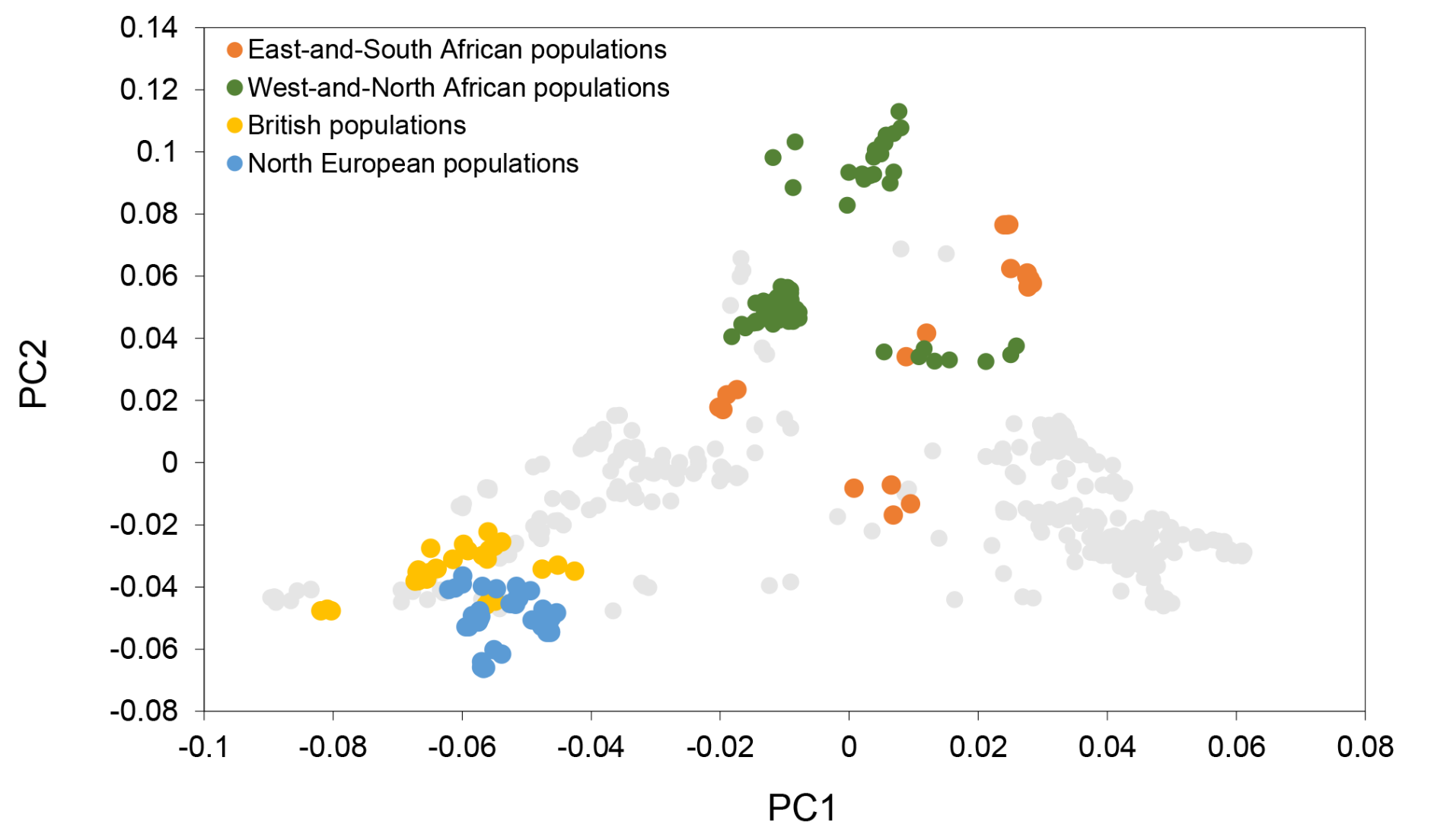


**Supplementary Fig. S11. Principal component analysis (PCA) highlighted East-and-South African, West-and-North African, British, and North European populations.**


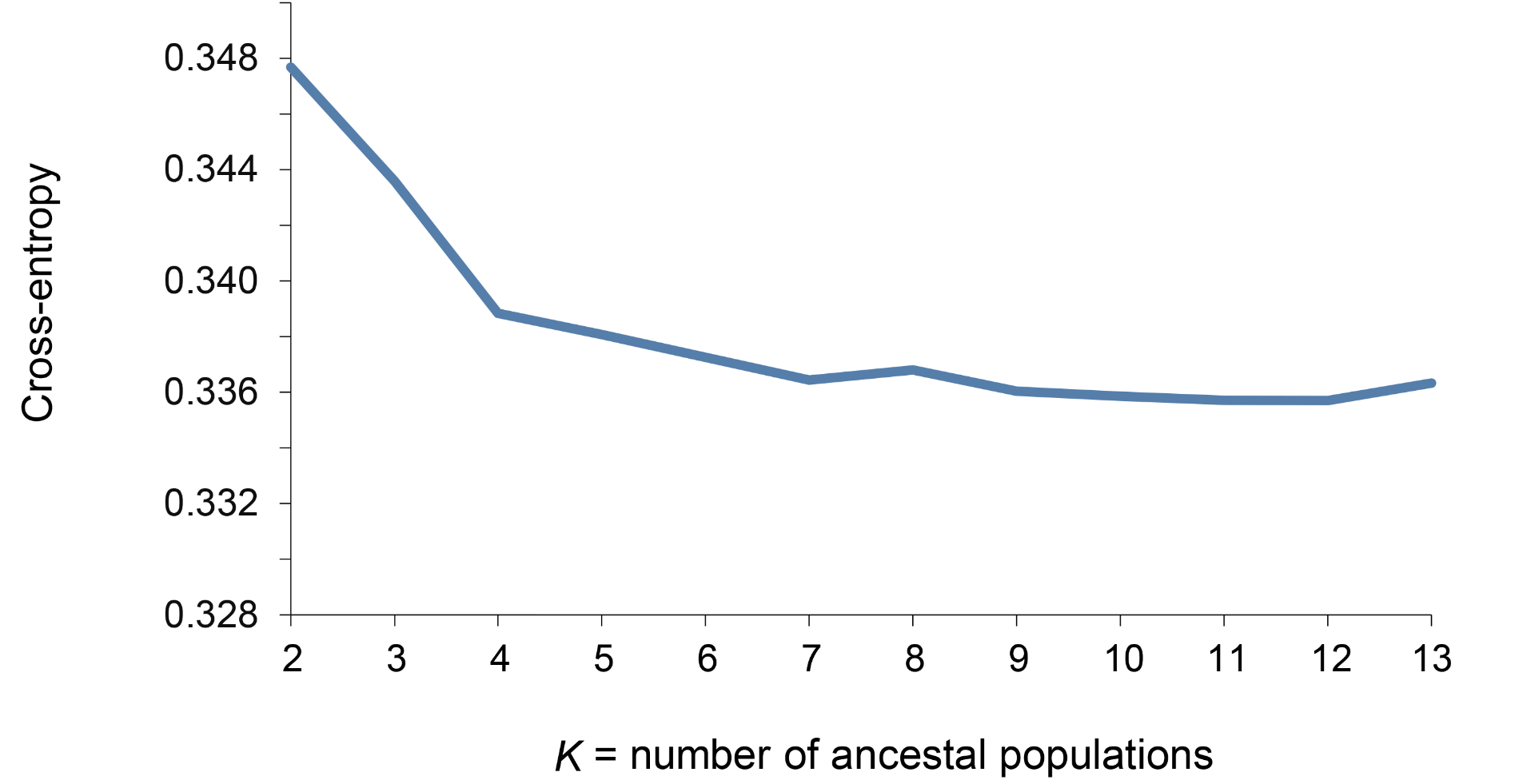


**Supplementary Fig. S12. Estimates of cross-entropy criterion for *K* = 13 sNMF runs (i.e., 13 ancestral populations).**


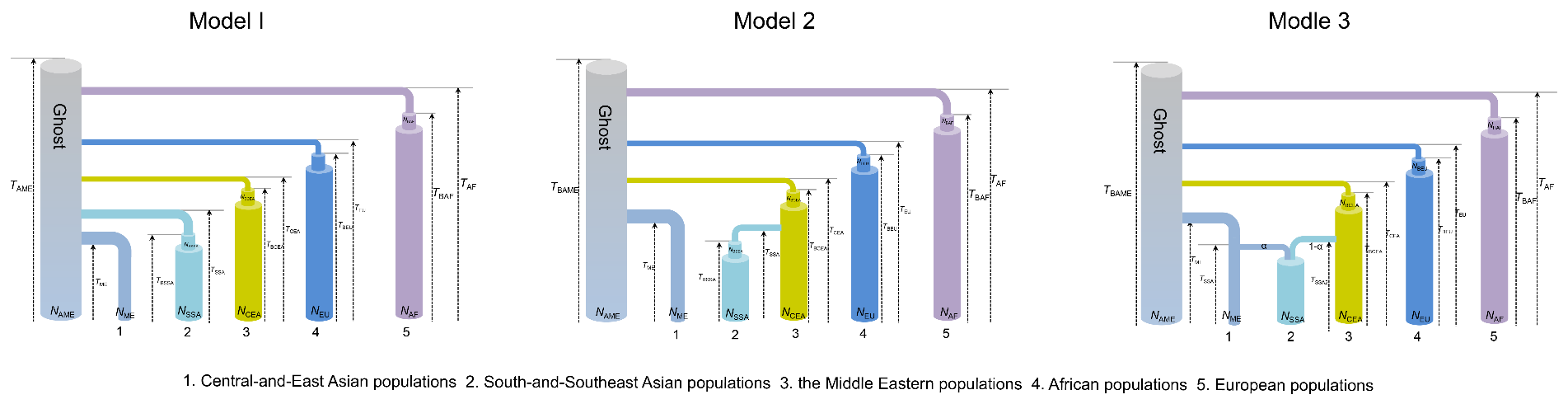


**Supplementary Fig. S13. Three alternative models for the demographic history of South and Southeastern Asian sheep without gene flow** **among groups of populations**. **(A)** Model 1: being descent of ancestral Middle Eastern populations; **(B)** Model 2: being derived from Central-and-East Asian populations; **(C)** Model 3: being originated from the early admixture between Central-and-East Asian populations and the Middle Eastern populations.


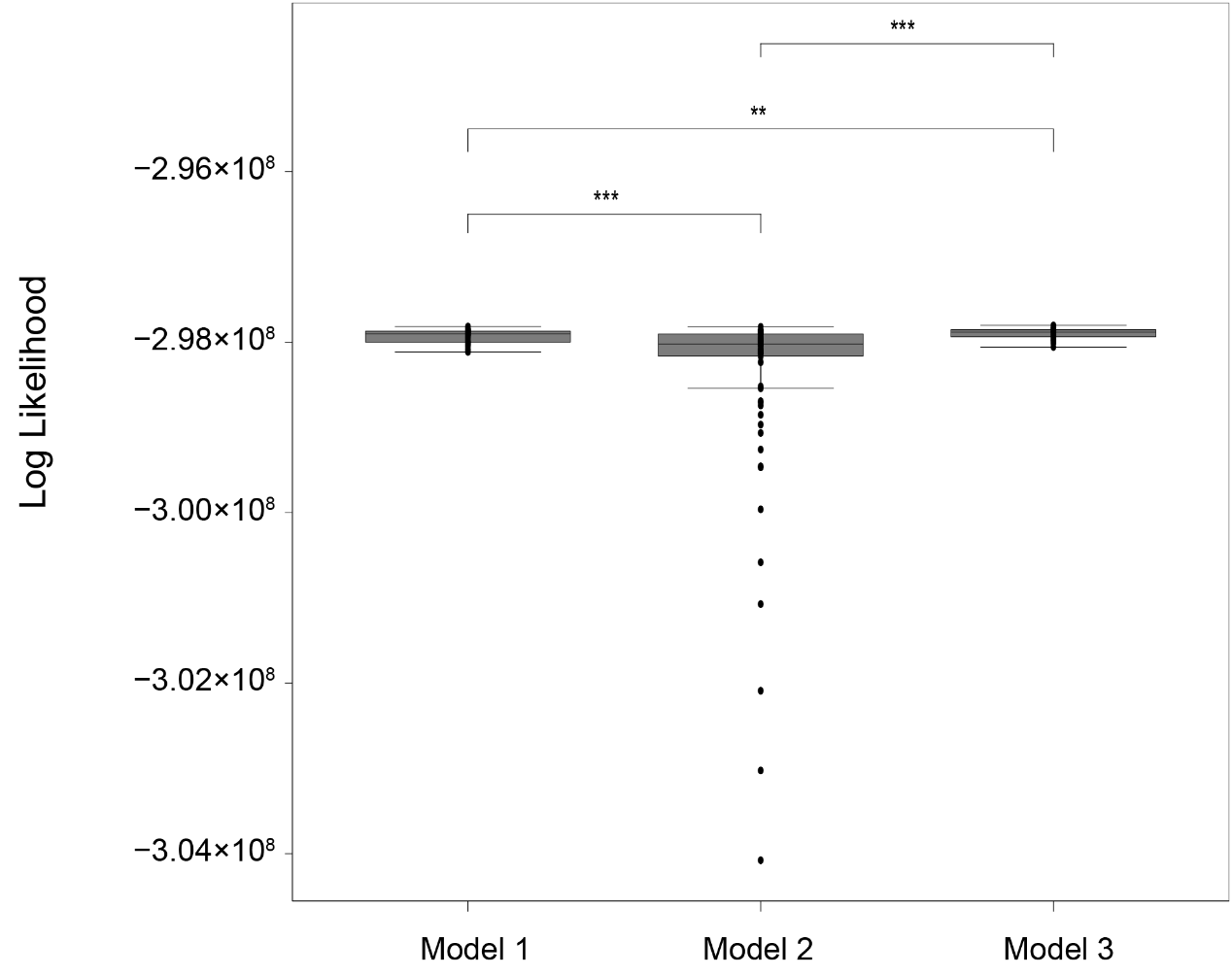


**Supplementary Fig. S14. Comparisons of log_10_ (likelihood) for the three alternative models.** Distributions were generated from 100 expected SFS computed with 10^6^ coalescent simulations. Estimates of log (likelihood) indicate that the Model 3 is the best model.


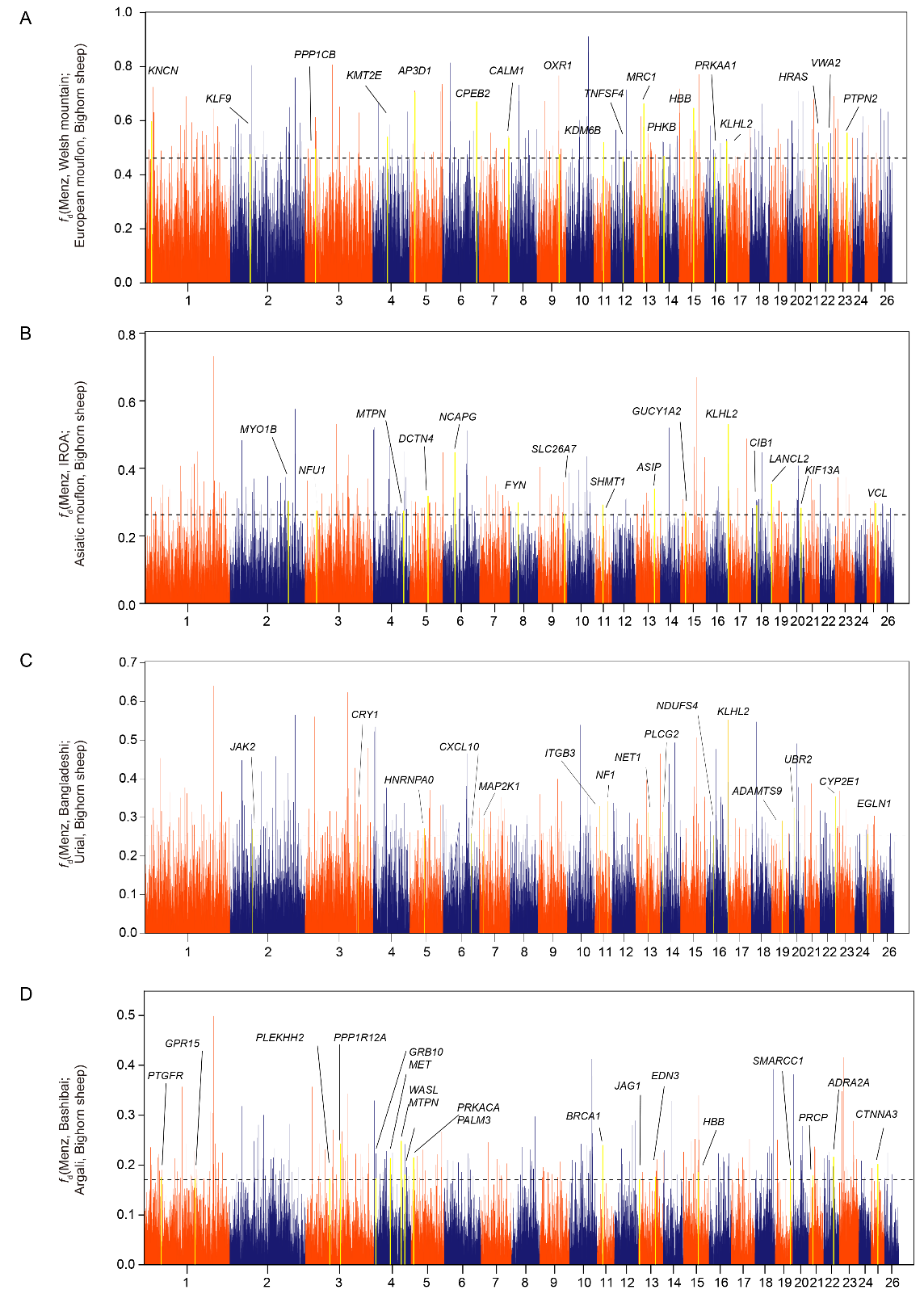


**Supplementary Fig. S15. Genome-wide distribution of *f*_d_ values**. **(A)** Across the genome estimated from the analysis of introgression from European mouflon to Welsh mountain sheep; **(B)** Across the genome estimated from the analysis of introgression from Asiatic mouflon to IROA (Iranian) sheep; **(C)** Across the genome estimated from the analysis of introgression from urial to Bangladeshi sheep; and **(D)** Across the genome estimated from the analysis of introgression from argali to Bashibai sheep.

**Supplementary Fig. S16. Enrichment analysis of functional genes identified by the introgression analyses**. GO and KEGG pathway enrichment analyses. Only significant (*P* < 0.05) GO terms and pathways and associated genes are shown.

**Supplementary Fig. S17. Signals of introgression in the hemoglobin (*HBB*) gens.** (A) Genetic divergence (*d*_xy_) between Asiatic mouflon and Changthangi sheep (orange line) or Menz sheep (blue line) is estimated across chromosome 15. (B) Pairwise *F*_ST_ values between populations (Changthangi sheep *vs.* Hu sheep, Changthangi sheep *vs.* East Friesian sheep, and East Friesian sheep *vs.* Hu Sheep) indicate divergences between the populations. (C) Patterns of haplotypes of *LOC114118424*, *HBE2*, *HBBC*, *HBE1*, *LOC114118425* and *HBB* genes in wild and domestic sheep.

**Supplementary Fig. S18. Mutation site of *GDF6* gene and the allele frequencies in population with different statures.** ARGS, argali; AMUF, Asiatic mouflon; EMUF, European mouflon; URIS, urial; SNOW, snow sheep; THNS, thinhorn sheep; BIGS, bighorn sheep; WAD, West African Dwarf sheep; KAG, Kage sheep; OUE, Ouessant; DJI, Djiallonke sheep.

**
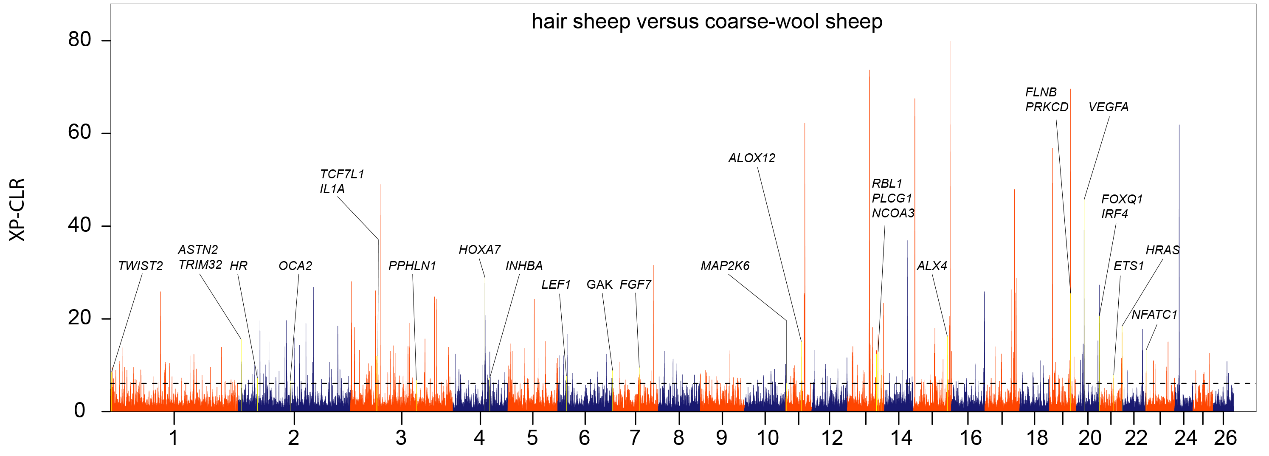
**

**Supplementary Fig. S19. Manhattan plot of selective signals associated with the fleece fiber variation between hairy and coarse-wool populations of domestic sheep by the Cross-Population Composite Likelihood Ratio (XP-CLR) method**.


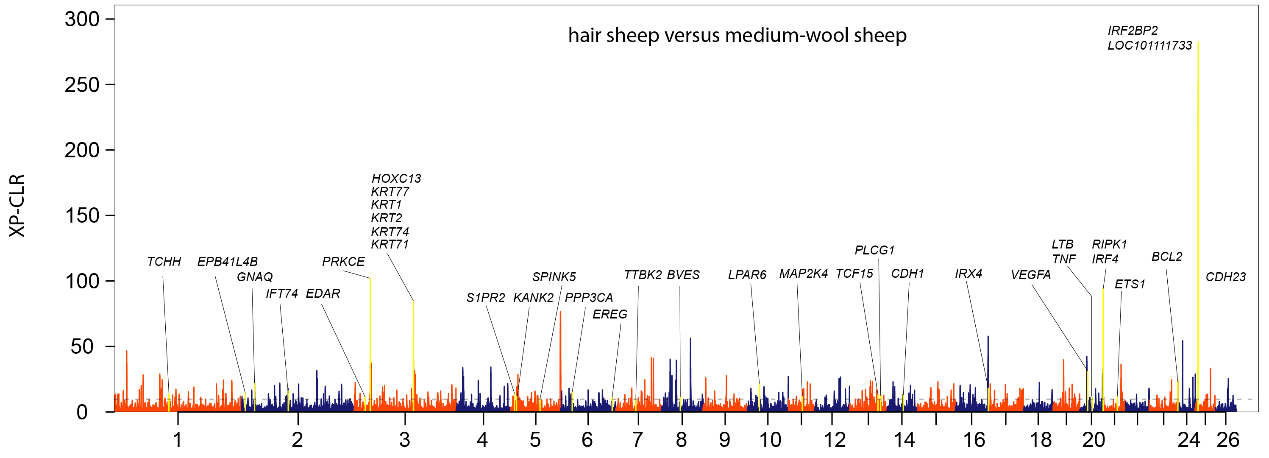


**Supplementary Fig. S20.** **Manhattan plot of selective signals associated with the fleece fiber variation between hairy and medium-wool populations of domestic sheep by the Cross-Population Composite Likelihood Ratio (XP-CLR) method**.


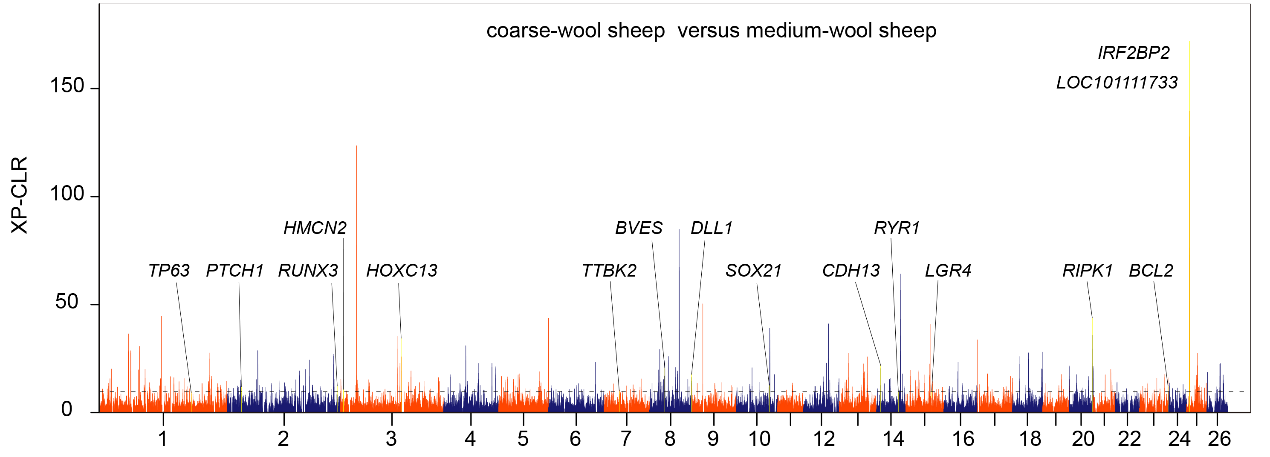


**Supplementary Fig. S21. Manhattan plot of selective signals associated with the fleece fiber variation between coarse-wool and medium-wool populations of domestic sheep by the Cross-Population Composite Likelihood Ratio (XP-CLR) method**.


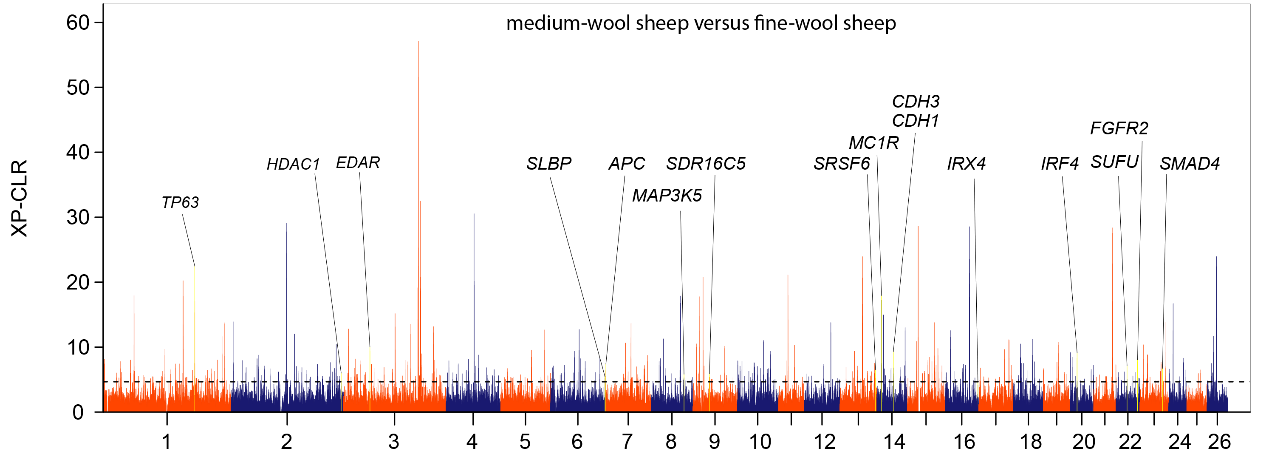


**Supplementary Fig. S22. Manhattan plot of selective signals associated with the fleece fiber variation between medium-wool and fine-wool populations of domestic sheep by the** **Cross-Population Composite Likelihood Ratio (XP-CLR) method**.


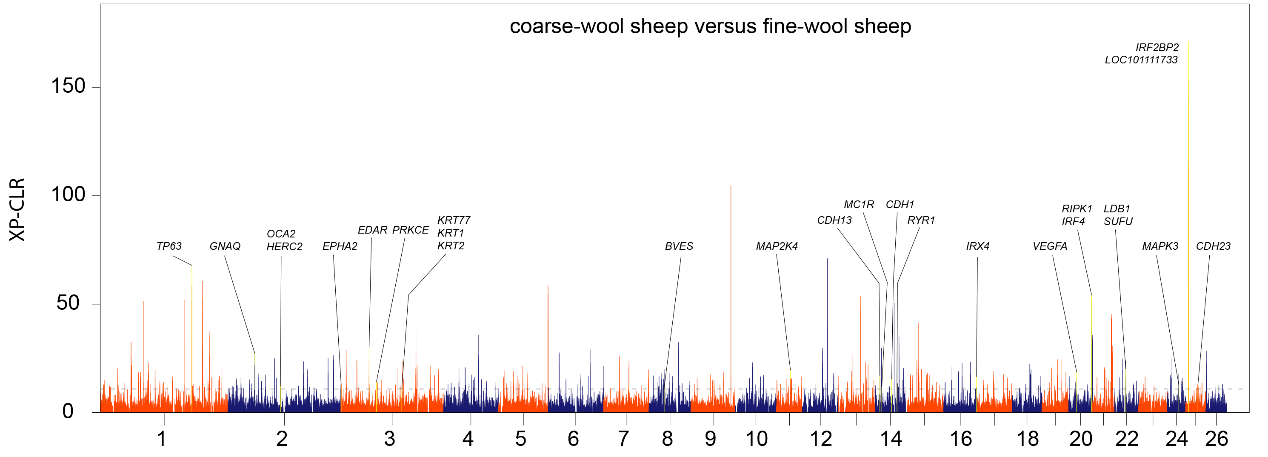


**Supplementary Fig. S23.** **Manhattan plot of selective signals associated with the fleece fiber variation between coarse-wool and fine-wool populations of domestic sheep by the Cross-Population Composite Likelihood Ratio (XP-CLR) method**.


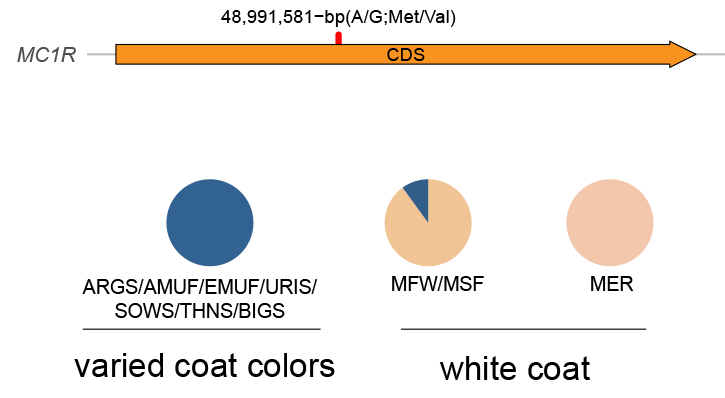


**Supplementary Fig. S24.** **Mutation site of *MC1R* gene and the allele frequencies in population with different coat colors.** ARGS, argali; AMUF, Asiatic mouflon; EMUF, European mouflon; URIS, urial; SNOW, snow sheep; THNS, thinhorn sheep; BIGS, bighorn sheep; MFW, Chinese Merino sheep (fine wool); MSF, Chinese Merino sheep (super fine wool); MER, Merino.


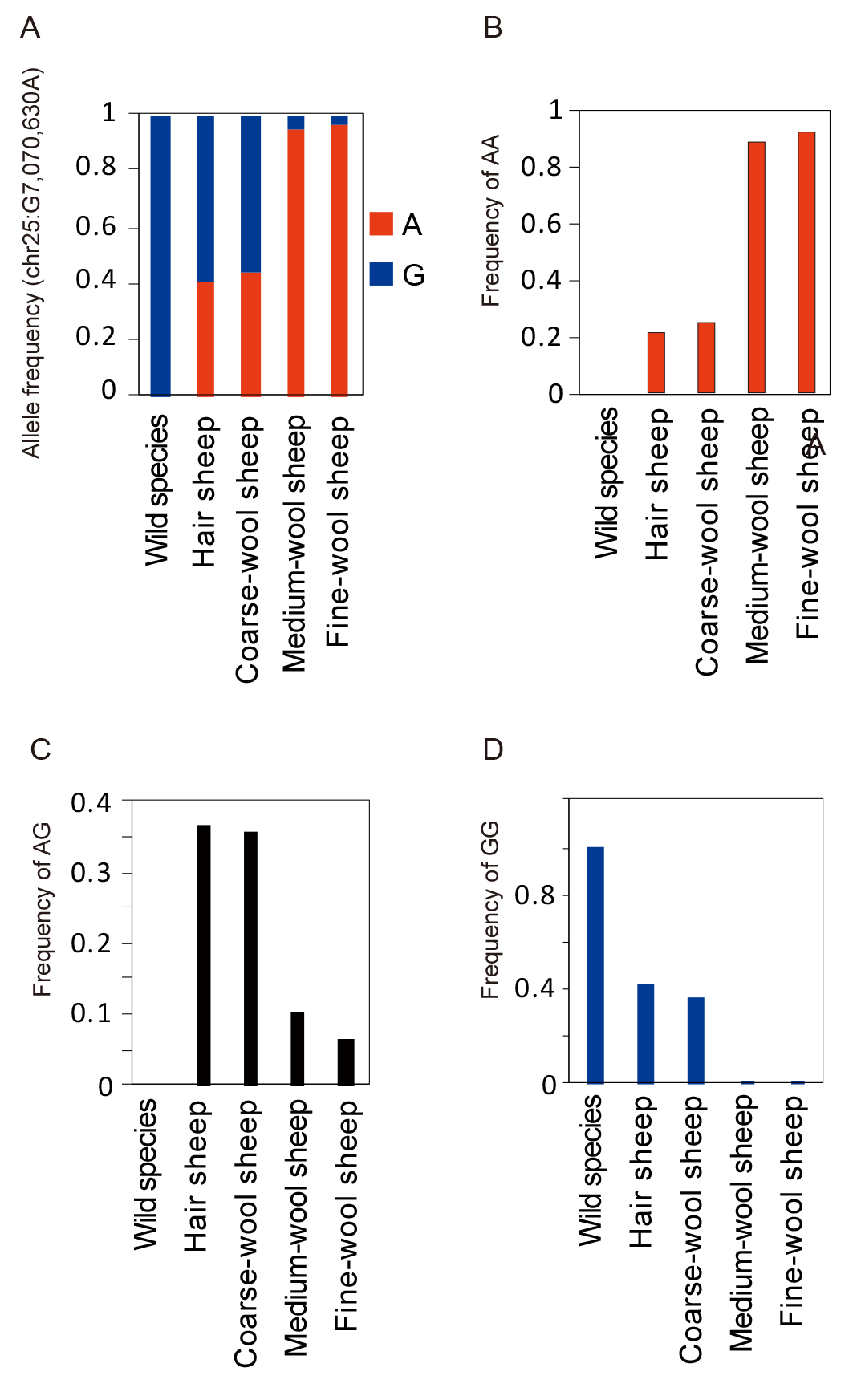


**Supplementary Fig. S25. Allele and genotype frequencies of for intron site (chr25:G7,070,630A)**. **(A)** alleles A and G, **(B)** genotype AA, **(C)** genotype AG, **(D)** genotype GG.

**
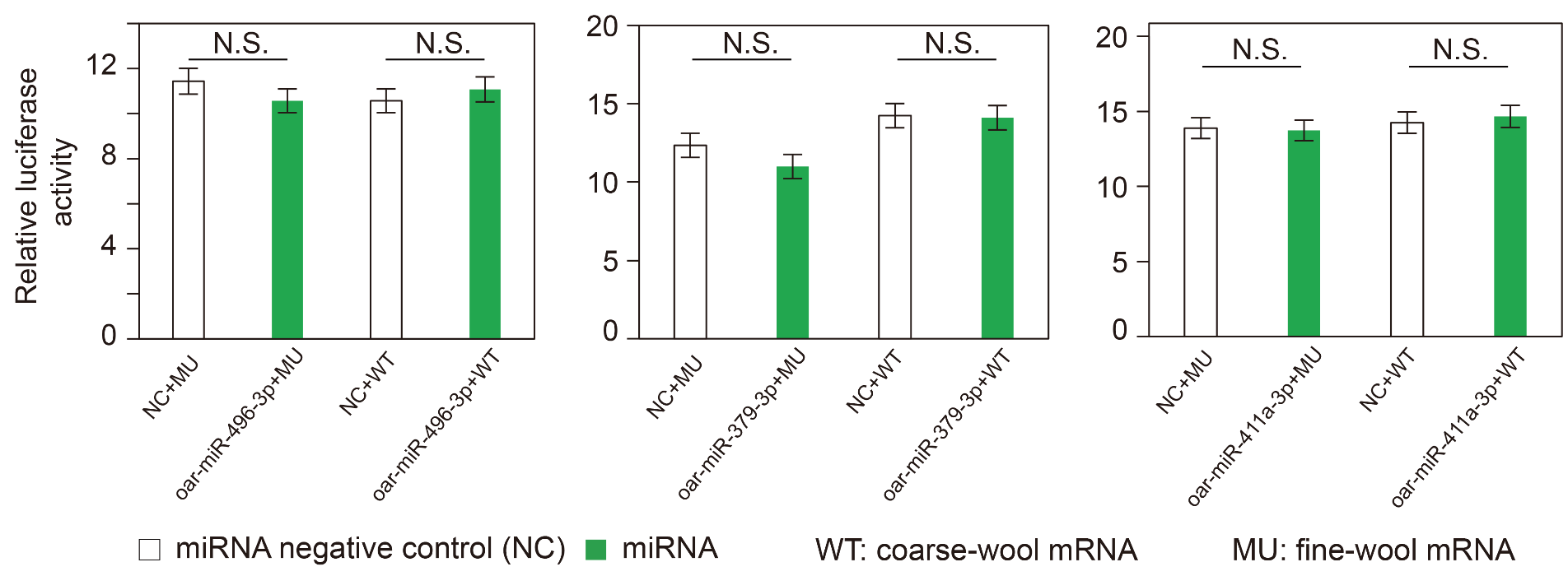
**

**Supplementary Fig. S26. Dual luciferase gene reporter assays for *IRF2BP2* gene and miRNAs such as oar-miR-496-3p (left), oar-miR-379-3p (medium), and oar-miR-411a-3p (right)**.


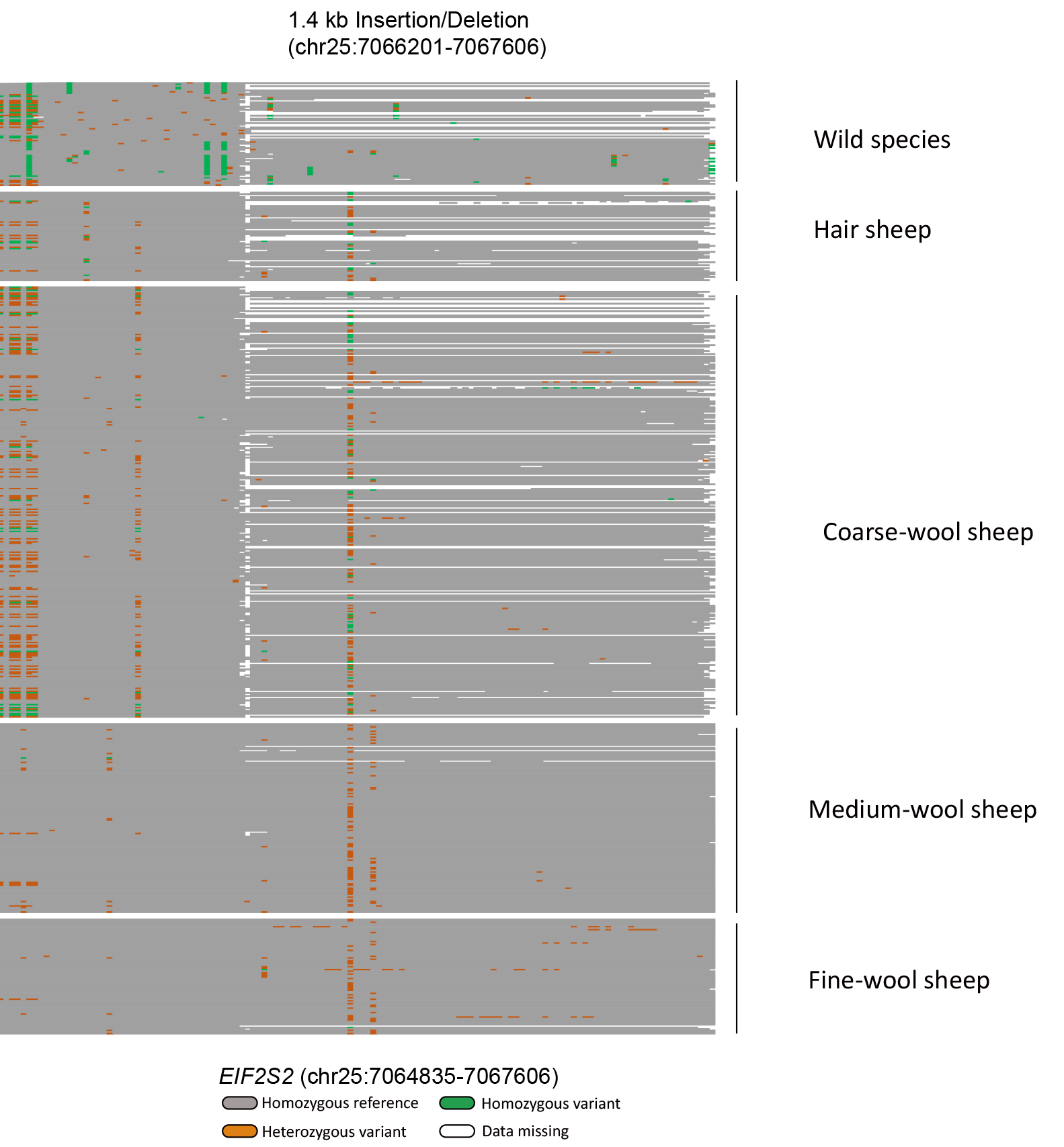


**Supplementary Fig. S27**. **The patterns of genotypes of *EIF2S2.***


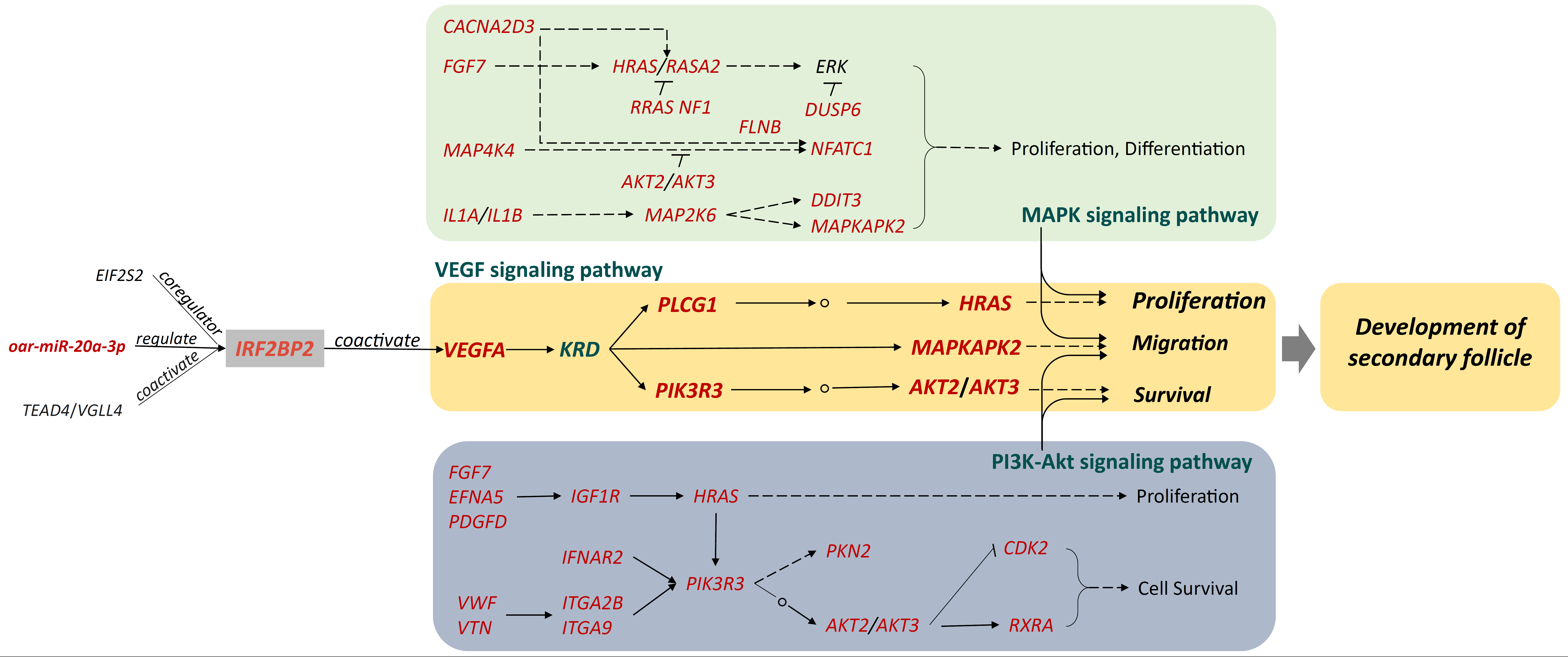


**Supplementary Fig. S28. Schematic mechanisms of signaling pathways with fleece fiber variation in domestic sheep**. The candidate genes identified by XP-CLR are indicated in red.


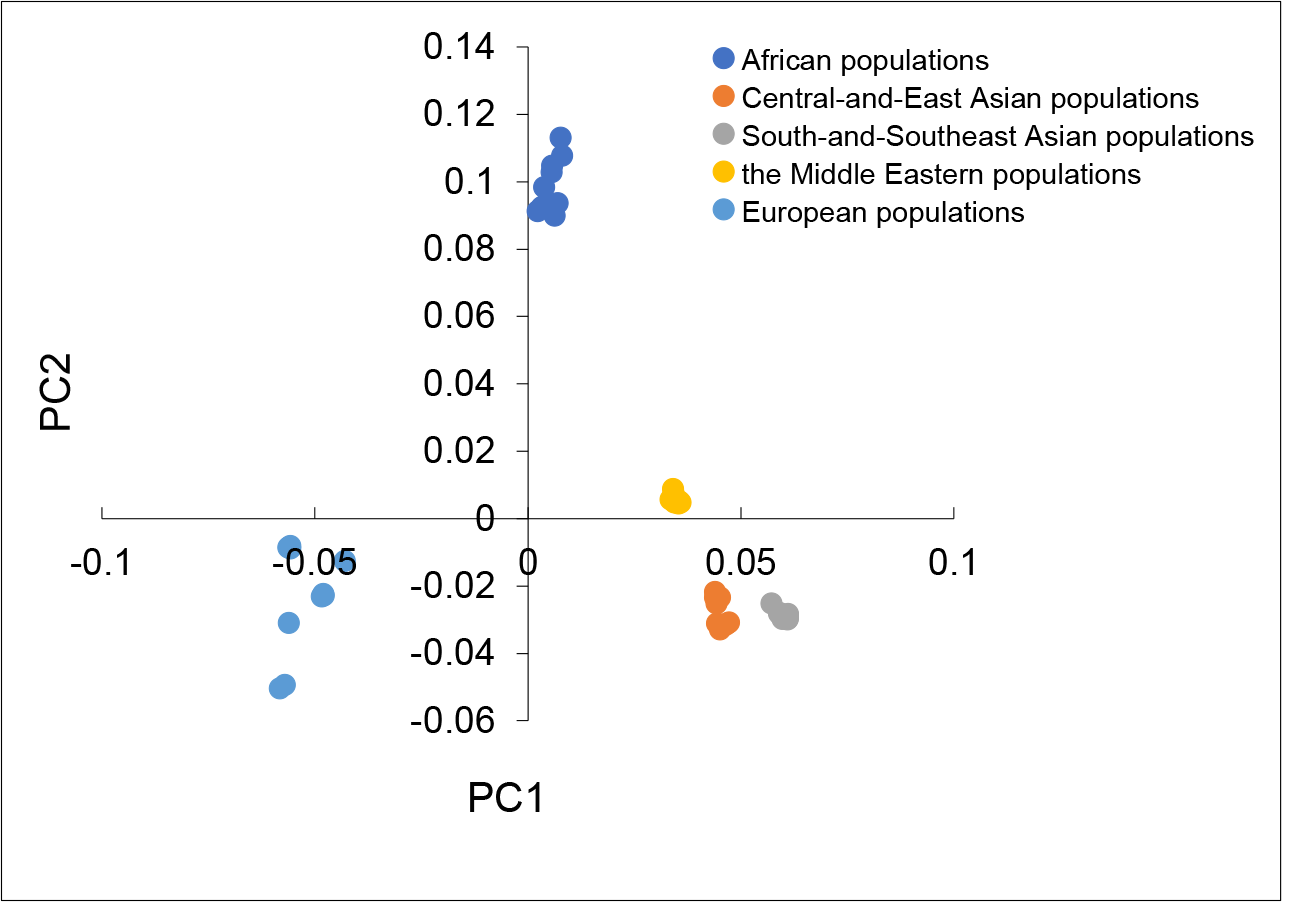


**Supplementary Fig. S29. Principal component analysis (PCA) of 48 individuals of domestic sheep included in the demographic reconstruction analysis.** The results indicate that the 48 individuals are representative of five geographic and genetic groups.

**
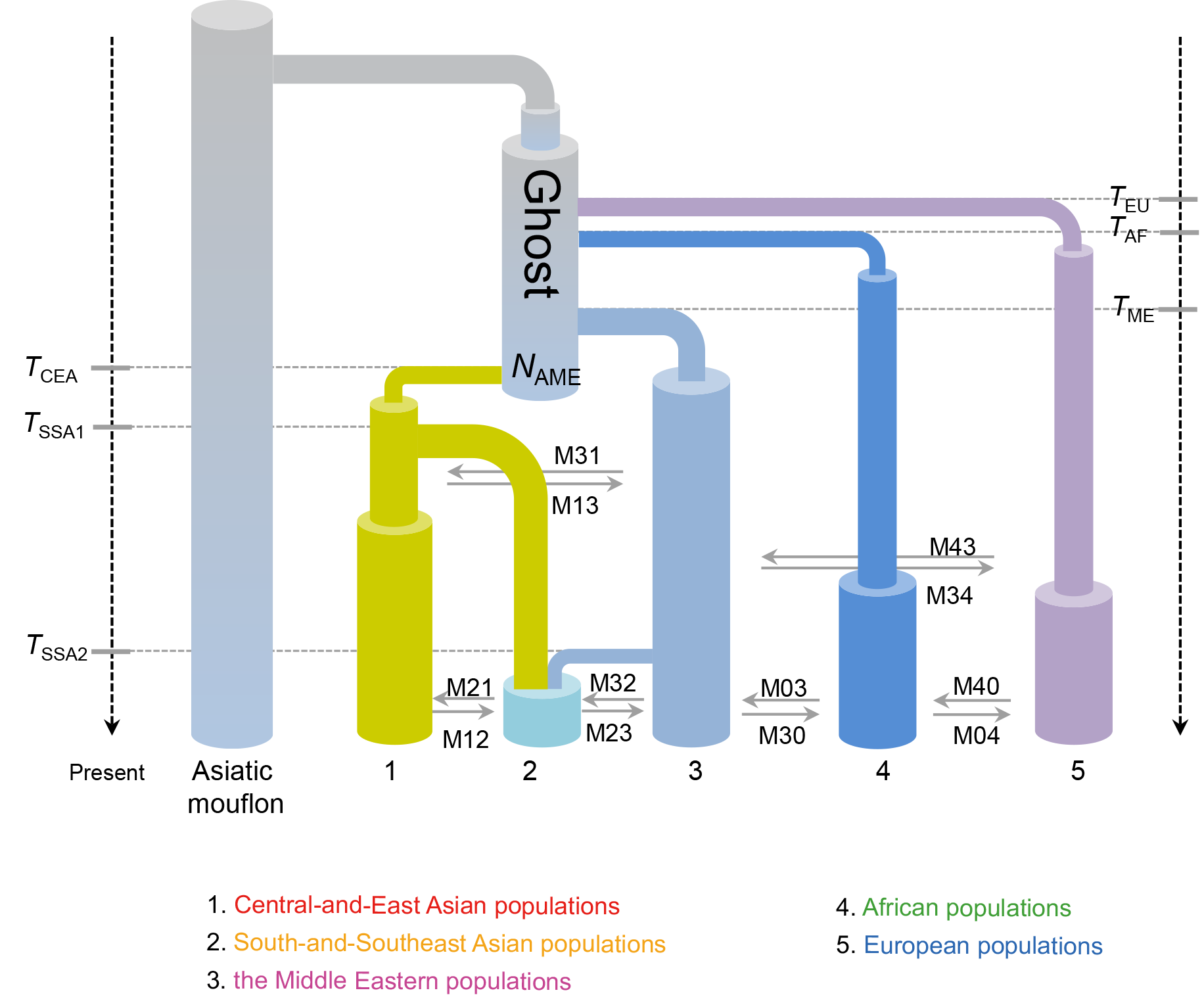
**

**Supplementary Fig. S30. Schematic representation of the best model 3 with gene flow.**


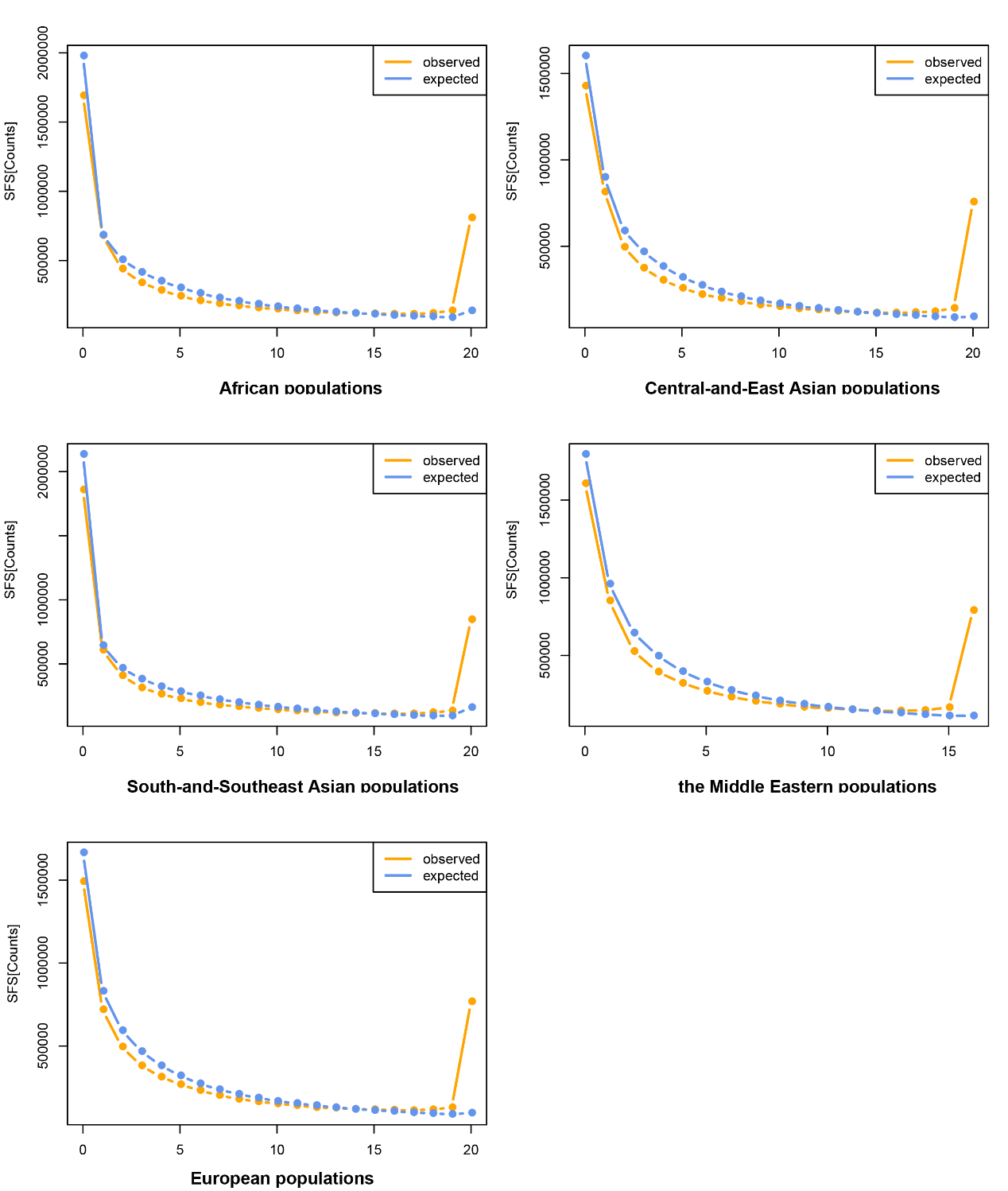


**Supplementary Fig. S31. Comparison of the marginal observed and marginal expected SFS for the best model 3**. The x-axis shows the derived allele frequencies (allele counts), while the y-axis shows the number of SNPs. The expected SFS was obtained from 100 SFS computed with 10^6^ coalescent simulations under the best model 3.

**Supplementary Tables (see the excel files)**

**Supplementary Table S1**. Summary information of domestic and wild sheep in this study.

**Supplementary Table S2**. Statistical significance of differences in the number of variants among species by the Mann-Whitney test.

**Supplementary Table S3**. The number of SNPs in domestic sheep populations from different geographic regions including shared and region-specific SNPs.

**Supplementary Table S4**. Validation of SNPs identified here in the *Ovis aries* dbSNP database (version 151).

**Supplementary Table S5**. Validation of SNP calling by Sanger sequencing.

**Supplementary Table S6**. Annotation of single nucleotide polymorphism (SNPs).

**Supplementary Table S7**. Annotated 3,592 functional genes associated with 6,816 SVs unique in domestic sheep.

**Supplementary Table S8**. Gene ontology (GO) enrichment terms and KEGG pathways (FDR *P*-value < 0.01) for 3592 candidate genes associated with 6,816 SVs unique in domestic sheep.

**Supplementary Table S9**. Gene ontology (GO) enrichment terms and KEGG pathways (*P*-value < 0.01) for 8,215 annotated candidate genes associated with 19,875 SVs unique in wild sheep.

**Supplementary Table S10**. Nucleotide diversity (π) of each domestic sheep population.

**Supplementary Table S11**. Nucleotide diversity (π) of the same domestic sheep in previous studies.

**Supplementary Table S12.** Nucleotide diversity (π) of each domestic sheep population in this and previously studies.

**Supplementary Table S13**. Kinship between samples with kinship coefficient larger than 0.0884 (the 2nd-degree relatedness).

**Supplementary Table S14**. Log-likelihood values obtained for the models reconstructed for five geographic groups of populations and a ghost population.

**Supplementary Table S15**. Point estimates and 95% confidence intervals for the parameters of the best mode (model 3).

**Supplementary Table S16**. Point estimates and 95% confidence intervals for the 2Nem migration rates for the best model (model 3).

**Supplementary Table S17**. *D*-statistics from wild species (argali, Asiatic mouflon, urial, European mouflon, snow sheep, thinhorn, and bighorn sheep) to domestic sheep populations.

**Supplementary Table S18**. *f*_d_ statistics from wild species (argali, Asiatic mouflon, urial, European mouflon, snow sheep, thinhorn, and bighorn sheep) to domestic sheep populations [*f*_d_ (Menz sheep, X; Wild sheep, Bighorn sheep), X indicate a candidate domestic population].

**Supplementary Table S19**. Proportion of retained or introgressed ancestry in domestic sheep populations from wild sheep species.

**Supplementary Table S20**. Details for chromosome-level introgression.

**Supplementary Table S21**. 79 common introgressed blocks from argali, urial, Asiatic mouflon, and European mouflon.

**Supplementary Table S22**. A total of 125 genes across the 79 common introgressed blocks from argali, urial, Asiatic mouflon, and European mouflon.

**Supplementary Table S23**. The Gene Ontology (GO) enrichment and KEGG pathway analysis for the 125 genes associated with 79 common introgressed blocks.

**Supplementary Table S24**. Population Branch Statistic (PBS) of putative selective regions in the Changthangi sheep population.

**Supplementary Table S25**. Putative genomic regions under selection based on the top 1% values of global *F*_ST_.

**Supplementary Table S26**. Overlaps regions between selected regions identified by global *F*_ST_ and QTLs reported previously.

**Supplementary Table S27**. Allele frequency of missense variants for the focused genes.

**Supplementary Table S28**. Putative regions under selection based on the top 1% values of deduction of diversity (ROD).

**Supplementary Table S29**. Details of SNP for nonsynonymous to synonymous (N/S) ratio (1508/2244=1.5) in the region of ROD peak (chr15:49,000,001-50,000,000).

**Supplementary Table S30**. Putative structure variations (SVs) under selection and candidate genes between landraces improved populations of domestic sheep.

**Supplementary Table S31**. Putative genomic regions under selection in the Ouessant population.

**Supplementary Table S32**. Wool fleece types (wild/hair/coarse-wool/medium-wool/fine-wool) for the samples studied.

**Supplementary Table S33**. Summary information on putative genomic regions with the top 1% XP-CLR values on each chromosome (hair *vs*. fine-wool sheep populations).

**Supplementary Table S34**. Summary information of putative genomic regions with the top 1% XP-CLR values on each chromosome (hair *vs*. coarse-wool sheep populations).

**Supplementary Table S35**. Summary information of putative genomic regions with the top 1% XP-CLR values and candidate genes on each chromosome (hair *vs*. medium-wool sheep populations).

**Supplementary Table S36**. Summary information of putative genomic regions with the top 1% XP-CLR values and candidate genes on each chromosome (coarse-wool *vs*. medium-wool sheep populations).

**Supplementary Table S37**. Summary information of putative genomic regions with top 1% XP-CLR values and candidate genes on each chromosome (medium-wool *vs*. fine-wool sheep populations).

**Supplementary Table S38**. Summary information of putative genomic regions with top 1% XP-CLR values and candidate genes on each chromosome (coarse-wool *vs*. fine-wool sheep populations).

**Supplementary Table S39**. Summary information of putative structure variations (SVs) under selection and candidate genes (hair *vs*. fine-wool sheep).

**Supplementary Table S40**. Summary information of putative SVs under selection and candidate genes (hair *vs*. coarse-wool sheep).

**Supplementary Table S41**. Summaries on putative SVs under selection and candidate genes (hair *vs*. medium-wool sheep).

**Supplementary Table S42**. Summary information of putative SVs under selection and candidate genes (coarse-wool *vs*. medium-wool sheep).

**Supplementary Table S43.** Summary information of putative SVs under selection and candidate genes (medium-wool *vs*. fine-wool sheep).

**Supplementary Table S44**. Summary information of putative SVs under selection and candidate genes (coarse-wool *vs*. fine-wool sheep).

**Supplementary Table S45**. Samples of sheep skin tissues for RNA-Seq analyses.

**Supplementary Table S46**. Details of the sequences in dual luciferase gene reporter assays.

**Supplementary Table S47**. Samples of sheep skin tissue for miRNA-Seq analyses.

**Supplementary Table S48**. Previous studies of genetic introgression in domestic animals.

**Supplementary Table S49**. Segregation of the asEIF2S2 and 3'UTR (chr25:7068586) mutations in wild and domestic sheep.

**Supplementary Table S50**. Samples used in the demographic modelling analyses.
